# Factors associated with recruitment to randomised controlled trials in general practice: a systematic mixed studies review

**DOI:** 10.1186/s13063-022-06865-x

**Published:** 2023-02-06

**Authors:** Keith R. Moffat, Wen Shi, Paul Cannon, Frank Sullivan

**Affiliations:** 1 Population and Behavioural Science Division, School of Medicine, Medical & Biological Sciences, North Haugh, St Andrews, UK; 2 College Librarian Medical, Veterinary & Life Sciences, Information Services, University of Glasgow Library, Hillhead Street, Glasgow, G12 8QE UK

## Abstract

**Background:**

A common challenge for randomised controlled trials (RCTs) is recruiting enough participants to be adequately powered to answer the research question. Recruitment has been set as a priority research area in trials to improve recruitment and thereby reduce wasted resources in conducted trials that fail to recruit sufficiently.

**Methods:**

We conducted a systematic mixed studies review to identify the factors associated with recruitment to RCTs in general practice. On September 8, 2020, English language studies were identified from MEDLINE, EMBASE, Cochrane Database of Systematic Reviews and CENTRAL databases for published studies. NTIS and OpenGrey were searched for grey literature, and BMC Trials was hand searched. A narrative synthesis was conducted for qualitative studies and a thematic synthesis for qualitative studies.

**Results:**

Thirty-seven studies met the inclusion criteria. These were of different study types (10 cross-sectional, 5 non-randomised studies of interventions, 2 RCTs, 10 qualitative and 10 mixed methods). The highest proportion was conducted in the UK (48%). The study quality was generally poor with 24 (65%) studies having major concerns. A complex combination of patient, practitioner or practice factors, and patient, practitioner or practice recruitment were assessed to determine the possible associations. There were more studies of patients than of practices or practitioners.

**Conclusions:**

For practitioners and patients alike, a trial that is clinically relevant is critical in influencing participation. Competing demands are given as an important reason for declining participation. There are concerns about randomisation relating to its impact on shared decision-making and not knowing which treatment will be assigned. Patients make decisions about whether they are a *candidate* for the trial even when they objectively fulfil the eligibility criteria. General practice processes, such as difficulties arranging appointments, can hinder recruitment, and a strong pre-existing doctor-patient relationship can improve recruitment. For clinicians, the wish to contribute to the research enterprise itself is seldom an important reason for participating, though clinicians reported being motivated to participate when the research could improve their clinical practice. One of the few experimental findings was that opportunistic recruitment resulted in significantly faster recruitment compared to systematic recruitment. These factors have clear implications for trial design. Methodologically, recruitment research of practices and practitioners should have increased priority. Higher quality studies of recruitment are required to find out what actually works rather than what might work.

**Trial registration:**

PROSPERO CRD42018100695. Registered on 03 July 2018.

**Supplementary Information:**

The online version contains supplementary material available at 10.1186/s13063-022-06865-x.

## Background

Randomised controlled trials (RCTs) require a sufficient number of participants to be adequately powered. This is necessary for a trial to answer the specific research question. The significant difficulties in recruiting participants are well established [1–3]. A review of all 151 RCTs funded by the UK National Institute for Health Research (NIHR) Health Technology Assessment (HTA) Programme between 2004 and 2016 showed that only 40% of RCTs recruited 100% of their original target; 63% recruited 80% of original target and around one-third of RCTs extended their period of recruitment to increase recruitment [1]. While the HTA review does not report the actual/target recruitment percentages for those studies in general practice, a survey of 34 trials in UK general practice found similar results with less than one-third recruiting to the original timescale [4]. Studies also regularly describe difficulties in recruiting individuals to general practice-based trials [5–7].

As a result of the challenges in recruiting participants, *methods to boost recruitment in trials*, was set as a priority research area by clinical trials units in the UK and the Lind Alliance [8]. More recently, in 2018, an RCT recruitment priority setting exercise (PRioRiTy) set its top ten research priorities. Two, relevant to this review, included *What are the barriers and enablers for clinicians…in helping conduct randomized trials* and *What are the key motivators influencing members of the public’s decisions to take part in a randomised trial* [9].

Other relevant systematic reviews have focused on interventions to improve recruitment to RCTs [10–12]. While this is clearly important, for our purposes, this would not be sufficient in identifying factors associated with recruitment out of the context of an intervention. For example, there are studies that retrospectively investigate reasons for poor recruitment which are of value but are not interventional by type [13]. Additionally, there are non-randomised studies of interventions (NRSIs) aiming to improve recruitment that were omitted by other reviews [12]. A Cochrane qualitative evidence synthesis of factors that impact recruitment to randomised trials in healthcare focused on potential trial participants’ experiences and perceptions of recruitment [14]. The Cochrane review did not investigate the perspective of the recruiter, e.g. general practices as a unit or general practitioners. A recent systematic review investigated NRSIs to improve participant recruitment [15]. The main finding from this was that all 92 included studies were at high risk of bias, and the authors concluded that *careful thought* is required for non-randomised studies to prevent further research waste.

Finally, none of these reviews focused on the specific setting of general practice but instead reported on recruitment in a wide range of clinical settings.

This systematic review focuses on recruitment to RCTs in general practice, also known as family practice or family medicine. The reason for this is that for many nations, care delivered in the community is the main paradigm of healthcare [16, 17] and which has increasing priority as societies attempt to deliver universal healthcare coverage [18]. Research findings from RCTs that have only been studied in a hospital setting cannot be assumed to generalise to a community setting. A robust evidence base of treatment effectiveness for people in the community is therefore required. As such, a research priority is recruitment in a general practice setting as recognised by the development of practice-based research networks internationally [19–21]. For this review’s purpose, both recruiters and participants are included.

A relevant area for further research is that of predictive modelling of recruitment to trials and the call for better models in predicting recruitment [22–25]. In 2020, The Tufts Center for the Study of Drug Development reported that 23% of studies recruited outside their target timeline. Although this was reduced from 52% in 2012, improved prediction of recruitment has been recommended to further reduce delays [26]. The Tufts’ approach is also supported by PRioRiTy where an identified priority included *What are the best ways to predict recruitment rates to a randomized trial and what impact do such predictions have on recruitment?* [9].

The aim of this systematic review is to identify the factors associated with the recruitment of individual patients, practices or practitioners to RCTs in general practice. In the longer term, we aim to develop a predictive model of RCT recruitment rates in general practice settings using machine learning methods and will use the findings from this review in selecting possible predictor variables from appropriate datasets.

## Methods

A completed PRISMA checklist for this review is provided in Additional file 1 [27].

### Protocol and registration

The protocol has been registered with The International Prospective Register of Systematic Reviews [28], (PROSPERO registration number: CRD42018100695) and published in BMC Trials [29].

### Eligibility criteria

Complete study eligibility is detailed in Table 1. In summary, we included any primary study design that investigated the recruitment of patient, practices, or practitioners to RCTs in general practice.

**Table 1:** Inclusion and exclusion criteria

Category	Inclusion criteria
Study design	Any primary study design that investigates the recruitment to RCTs in general practice. Qualitative, quantitative and mixed methods studies were included.
Participants	Any participants, both where the focus was on recruitment of practices to RCTs as well as recruitment of individual patients.
Interventions	Any interventions that target recruitment.
Comparators	Any comparators were included.
Outcomes	Outcomes that are focused on recruitment. Examples of outcomes include the number of participants recruited, percentage of recruitment target achieved and time to the first participant recruited.
Setting	The study investigating recruitment must be of any RCT that is set within general practice, i.e. general practices are the locus of the intervention where recruited participants are randomised to an intervention based on the practice.
Language	Only studies in English were included.

### Information sources

The PRISMA-S checklist was used to describe the literature search process [30].

The following databases were searched for relevant studies: MEDLINE In-Process and other Non-Indexed Citations and MEDLINE 1946 to Present; Embase 1947-Present, updated daily, both via Ovid; the Cochrane Database of Systematic Reviews, Issue 5 of 12, May 2018; and the Cochrane Central Register of Controlled Trials (CENTRAL), both via the Cochrane library. These databases were searched on 23 May 2018. In addition, the websites OpenGrey [31] and the National Technical Reports Library [32] were searched on 1 June 2018. All searches were updated on 8 September 2020. The journal BMC trials were hand-searched, originally for 5 years preceding the 23rd of May 2018. This hand search was extended to 8 September 2020 to align with the updated database search. Hand-searching was conducted by looking back through the articles section of the BMC trials website [33]. No filtering was applied to this.

Experts in the field of trial recruitment were contacted regarding important articles for inclusion.

### Search strategy

The search strategy was supported by a health information specialist with systematic review experience (PC). The search strategies used both text words and relevant indexing related to controlled or multicentre trials, selection and recruitment, and general practices and practitioners. Citation and bibliographic searching were conducted on any included studies to identify additional relevant studies. A snowballing process was used for any studies discovered where further bibliographic and citation review was repeated until no further papers were found [34]. Citation searching was done by using the cited by section of PubMed for each article [35]. Bibliographic searching was done by using reference lists at the end of each article. The full search strategies are attached separately in Additional file 2.

### Study records

Literature search results were exported to the DistillerSR systematic review software. Based on the inclusion and exclusion criteria (Table 1) the review team developed screening questions and forms that were tested by a calibration exercise which involved reviewing a sample of level 1 and 2 screening with a 3rd reviewer (FS) prior to implementation. These were uploaded to DistillerSR along with citation abstracts and full articles. Any members of the review team that were not familiar with DistillerSR or the subject area received training.

**Fig. 1:**
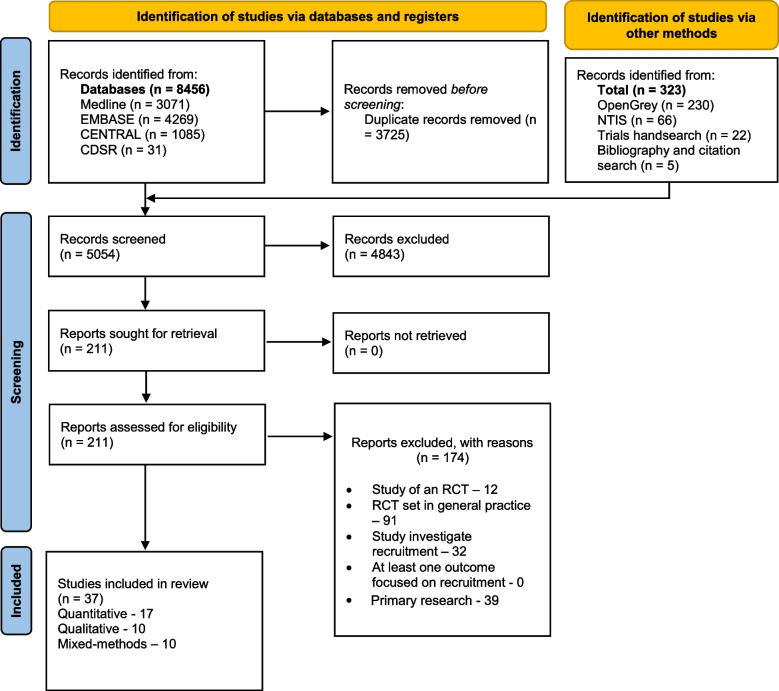
PRISMA flow diagram. This figure illustrates the number of studies identified at each stage of the screening process. RCT, randomised controlled trial

A PRISMA diagram [27] was completed to show the selection of studies at different levels of assessment and is shown in Fig. 1 of the results section.

### Selection process

Titles and abstracts of studies of search results and other sources were independently screened by two reviewers (KM and WS). The full text of those studies that potentially met the inclusion criteria was retrieved and independently assessed for eligibility by two reviewers. Any disagreements over the eligibility of studies were resolved through discussion with a third reviewer (FS). Reasons for excluding studies were recorded. The reviewers were not blinded to the journal titles, study authors or institutions.

### Data collection process

Standardised forms were used to extract data and were specific for quantitative, qualitative and mixed-methods studies. Two review authors (KM and WS) extracted data independently from included studies, and conflicts were resolved through discussion. Any disagreements were resolved with a third reviewer (FS). Extracted data included demographic information, methodology and outcomes as well as measured recruitment metrics. Study authors were contacted in the event of any incomplete data.

### Outcomes

The primary outcome was study recruitment. There are many ways of measuring this including numbers, proportions and rates.

For qualitative studies that investigated recruitment to trials, the *phenomenon of interest* [36] rather than outcome is used and that was recruitment.

### Quality appraisal

Two reviewers independently assessed study quality (KM and WS) at the study level. Since any study type was included in this systematic review, a variety of quality appraisal tools were used (Table 2).

**Table 2:** Quality appraisal tool used in this systematic review

Type of study	Quality appraisal tool used
**Quantitative**	
Randomised controlled trial	Cochrane collaboration risk-of-bias tool for randomized trials (RoB 2) [37]
Cross-sectional	Appraisal tool for Cross-Sectional Studies (AXIS) [38]
Case-control	Critical Appraisal Skills Programme (CASP) case-control study checklist [39]
Non-randomised studies of interventions, including time-series analysis	Cochrane Risk Of Bias In Non-randomised Studies of Interventions (ROBINS-I) [40]
**Qualitative**	CASP Qualitative Study Checklist [39]
**Mixed methods**	Mixed methods scoring system [41]

Risk of bias tools was used to categorise the risk of bias into low, moderate, serious or critical based on the algorithms contained in the full guidance documents for RoB 2 [42] and ROBINS-I [43].

For CASP, AXIS and mixed-methods tools, papers were judged: no concerns, if no element of the design was judged to be poor; minor concerns for one poor score; and major concerns for two or more poor scores.

The quality appraisal information was tabulated along with the corresponding studies and informs the discussion of the quality of the evidence base on this topic.

### Data synthesis

A narrative synthesis of quantitative studies was performed. Guidance from the Centre for Reviews and Dissemination informed the synthesis [44].

A thematic synthesis was conducted for qualitative studies, including qualitative components of mixed methods studies. This followed the three stages of thematic synthesis described by Thomas and Harden: *line-by-line* coding of text, development of *descriptive themes* and generation of *analytical themes* [45]. Study reports were entered verbatim into the NVivo qualitative data analysis software. One reviewer (KM) coded the text, developed descriptive themes and generated analytical themes. These were reviewed by a second reviewer (FS).

Since recruitment studies could investigate recruitment at different levels, outcome variables were categorised according to the recruitment focus, i.e. patient, practitioner and practice. Independent variables were also categorised by patient, practitioner and practice. Some studies investigated overlapping outcomes and factors. These were placed in separate analyses according to the relevant category. Where necessary, simple data conversions were performed to provide study size numbers and percentages where these were not explicitly stated. Studies were tabulated by outcome category, with subcategories or subthemes displayed within these. Certainty in the body of evidence for an outcome was assessed by reviewing quality appraisal results along with consistency with other studies reporting on the same recruitment factor. Findings provided in the discussion section are those where there is a high degree of confidence.

This mixed studies review used a parallel result convergent synthesis design which is characterised by quantitative and qualitative evidence being analysed and presented separately and integration occurring in the discussion section during the interpretation of results [46].

## Results

### Literature search

A PRISMA flow diagram (Fig. 1) shows the screening flow.

The database searches, *Trials* journal hand search and full-paper bibliography and citation search resulted in 7297 papers. A total of 3275 of these were duplicates. Title and abstract screening excluded 3810 papers leaving 212 full-text articles. A total of 167 of these were excluded. A total of 37 studies, therefore, met the inclusion criteria [7, 47–82].

### Description of included studies

A summary of the recruitment study characteristics including contextual information on the parent RCT is provided in Additional file 3.

#### Study participants

Of the 37 included studies, 27 investigated individual patient factors associated with recruitment, 6 investigated practice factors, 10 investigated practitioner factors and 12 investigated overlapping categories.

The study size was calculated according to the recruitment focus. For example, where patient recruitment was the focus, study size relates to the number of patients recruited and does not include a number of practices or practitioners even if factors related to these were used as independent variables.

In total, the studies involved 18,645 patients (13,098 participants, 5547 non-participants), 1173 practitioners (1004 participants, 169 non-participants) and 1335 practices (585 participants, 750 non-participants). There is an almost 20-fold higher number of patients compared with practitioners and practices.

#### Types of trials

The geographical locations of the studies were UK, 18; USA, 5; Australia, 5; New Zealand, 3; The Netherlands, 3; Germany, 1; Norway, 1; and Switzerland, 1.

The design of recruitment studies was varied. Quantitative studies included 10 cross-sectional design, 5 NRSIs and 2 RCTs. There were 10 qualitative studies and 10 mixed methods studies.

The clinical domains of the interventions were categorised as allergy 1, cancer 1, cardiovascular 6, care of older adults 2, dermatology 1, diabetology 1, gastrointestinal 2, haematology 1, infectious disease 3, medically unexplained Symptoms 1, mental health 2, musculoskeletal 4, obstetrics 2, occupational health 1, paediatrics 1, public health 6, respiratory 5 and renal 1.

#### Methodological limitations of the studies

Quality appraisal assessments of all studies are provided in Additional file 4 and graphical summaries in Additional file 5. We assessed 6 studies as having no methodological limitations (1 cross-sectional; 4 qualitative; 1 mixed-methods), 5 minor (2 RCTs; 1 cross-sectional; 1 qualitative; 2 mixed-methods), 2 moderate (both cross-sectional) and 24 as major (7 cross-sectional; 5 non-randomised studies of interventions; 5 qualitative; 7 mixed-methods). Studies were, therefore, generally of low quality. The results of quality appraisal should be considered along with the type of study which has clear implications on the level of evidence.

#### Heterogeneity

There was substantial heterogeneity across the studies with variation in study type, setting and definition of variables and outcomes. For example, studies defined ‘recruitment’ differently. Studies varied in their focus on recruitment of practices, practitioners or participants to trials. The type of factor also varied and could be a practice, practitioner, patient or trial factor.

### Factors associated with recruitment

Factors are divided into two main categories: factors associated with the recruitment of patients and factors associated with the recruitment of practices and/or practitioners. Within each of these categories, there are multiple subcategories including patient, practitioner, practice and trial factors. A summary of the results for each individual factor for quantitative and qualitative studies is provided in Additional files 6 and 7.

### Factors associated with recruitment of patients

#### Patient factors

##### Demographic factors

Age: Older age was consistently associated with poor recruitment in the four studies which investigated this [47, 60, 63, 80]. Importantly, these studies were all in the over 65 age group.

Sex: There were inconsistent findings for the association between male or female sex and recruitment. Two studies investigated this and had conflicting results [63, 80].

Deprivation: Fletcher et al. found a significant positive association between recruitment and the most deprived quartile [47]. Rogers et al. showed the opposite association with the highest number and percentage of participants in the least deprived quintile [80]. However, in the latter trial, few patients were invited from deprived background so no clear conclusion can be drawn.

Ethnicity: The findings from two studies reflect the higher number of white people eligible to studies, invited and therefore recruited [47, 70]. One of these studies, however, found that for some groups, the rates of invited to recruited was different with no Hispanic or Asian patients recruited from the 23 eligible [70].

Migrants: Welsh et al. found that immigrants were significantly less likely than non-migrants to be recruited with only 12% agreeing to recruitment [70].

##### Medical factors

Comorbidity: This association is unclear with Durham et al.’s finding that participants had a significantly lower chronic disease score (2.7), compared to non-participants (2.8). Fletcher et al. used the Rankin disability score [47] and did not show a clear association between disability score and recruited rate.

Polypharmacy: Two studies investigated the association with being prescribed five or more medications and found a small positive association with recruitment [47, 60]. One study was statistically significant [60].

Desire not to have medication altered: Petty et al. found that some patients did not want a change in their usual medications [60].

Health status can affect potential participation due to current health status, potential personal health benefits and fear of health risks: Petty et al. found that a number of patients stated they were not well enough to participate [60]. Normansell et al. found that there were different reasons that one’s health status could impact their reason for declining participation [52]. One patient had a history of stroke that affected their mobility and therefore the ability to take part in an exercise trial, while another had multiple health problems creating medical burden. Rogers et al. found that *For some, psychological barriers such as lack of confidence and depression posed significant challenges to participation* [80].

Van Staa et al. found that for six out of ten patients, the most important reason for participating in the eLung trial was that it might improve their own health [61]. Bleidhorn et al, also found that experience of personal health benefit from taking part in a previous trial encouraged them to take part in their trial [82].

Bleidhorn et al. found that some declined participation due to concerns that their health would be adversely impacted by taking part [82]. Rogers et al. found that *Real and perceived medical problems and fear of such problems were significant barriers to regular physical activity* [80].

##### Practical factors

Perceived time constraints and conflict with other commitments can serve as a negative influencing factor in considering participation: Attwood et al. found that barriers related to time—generally work and/or family commitments—were commonly raised by non-participants [69]. This functioned both as a perceived barrier to them participating in research generally but also in specifically finding the time to increase their activity levels. For participants interviewed by Bleidhorn et al., there was an assumption that people did not take part due to *trial-related time effort*, especially for employed people [82]. Rogers et al. also found that some had many other commitments relating to work, family and recreation [80]. It was also felt that the 3-month commitment to the trial was too long. Likewise, Normansell et al. found that the duration of their trial was a perceived barrier [52]. Normansell et al. also found that three (10%) respondents declined participation due to work or other commitments [52]. Caring commitments were also found to deter participation. Three (10%) respondents did not participate due to travelling away.

Travel difficulties: Normansell et al. found one potential participant declined due to difficulty with transport [52].

Unavailability: Petty et al. found that a number of patients declined participation due to unavailability for a number of reasons, including going on holiday, in-hospital or day centres, interference with their routine and moving away from the area [60].

Communication: Welsh et al. found a non-significant association with patients requiring a translator less likely to be recruited [70].

##### Beliefs

Not considering oneself a *candidate* for the trial can negatively influence the decision to participate: The concept of candidacy was originally described by Davison et al., as an explanatory framework of personal beliefs regarding coronary risk [83]. Individuals develop an idea of the *kind of person who gets heart trouble*, and this can impact the effectiveness of health promotion campaigns. Candidacy functioned as a *lay epidemiology* [84]. Other studies have researched candidacy beyond this strict definition. Tookey et al. found that the doctor-patient relationship could serve as both a *facilitator of candidacy* and a *barrier to care*, when patients are considering accessing general practice for cancer symptoms [85]. In this review, candidacy appears to be a useful framework in considering patients’ perceived eligibility to participate in trials. Attwood et al. interviewed non-participants to a trial with a pedometer-based intervention to identify reasons for non-participation [69]. Patients often felt they were not candidates for the trial due to health conditions or a disability. Additionally, based on their own views of their current health or activity levels, they would often decline, *I already do run about four times a week and cycle to and from work…and do Pilates, so I probably wouldn’t increase it. I felt like probably I wasn’t the ideal candidate*. Similarly, Rogers et al. found that many thought the trial was unnecessary for them because they perceived that their activity levels were high enough [80]. In the same study, some non-participants did not participate due to what they described as ‘personal competence’. Either they were competent enough with physical activity or due to health problems they would not be competent. Petty et al. found that some patients thought that due to the *perceived simplicity* of their medication regimen they *would be wasting the pharmacist’s time* [60]. Normansell et al. found that a reason for declining participation was a perception that the trial was intended for an older age group than theirs [52]. This highlights the importance of conclusions that potential participants will make about their own perceived candidacy for a trial, even if they would be objectively eligible based on the entry criteria.

Negative perceptions of research can influence participation in a variety of ways [82]. Bleidhorn et al. found that participant respondents felt that uncertainty over the effectiveness of the intervention and the perception of being a *test subject* experimented on could negatively influence participation. Participants also felt that non-participants did not value research as a potential reason for not participating. One interviewee who had participated thought that the negative media portrayal of research is a potential barrier for some.

Mistrust of the study: Petty et al. found that several patients were concerned that they would have medicines stopped that they were happy taking or that the study was designed to save money [60].

Attitude toward healthcare: Petty et al. found that some patients did not like contact with the healthcare system and did not consent as a result [60].

Age: Rogers et al. found patients accepted that in retirement they would be *slowing down, I find an awful lot of people our age, they think about exercise but very few do anything about it. As they get older, they slow down and can’t be bothered* [80].

##### Behaviours

Altruism is an important factor for some in deciding to participate: Bleidhorn et al. found that several patients were motivated to take part in the trial out of consideration for others [82]. This motivation was at two levels. One was to help research in general, and the other was to help women specifically affected by urinary tract infection (UTI). The general motivation was reflected in another qualitative study by van Staa et al. showing that four out of ten patients who took part in the study stated the main reason for doing so was to improve other people’s health in the future [61].

Spontaneity: Bleidhorn et al. found that some participants stated that they were open and curious and did not consider participation for long before consenting [82].

Existing routine: Rogers et al. found that in the context of an exercise intervention, behavioural change was resisted due to potential participants’ existing routine [80].

##### Research experience

Participants in Bleidhorn et al.’s study had previous experience in a trial and decided to participate because of this [82].

##### Attitudes

Gratitude: One respondent to Bleidhorn et al. agreed to participate due to being grateful for getting a last-minute appointment with a nurse for the assessment of their UTI [82].

#### Practice factors

##### Demographic

Age profile of practice population: A study aiming to recruit participants over the age of 75 found no significant difference between the proportion of the practice population over 75 and the percentage of the eligible population recruited [71].

Deprivation: Williams et al. found that high socioeconomic status (SES) was significantly associated with lower recruitment rates when compared with low SES [75].

Distance to travel: Brealey et al. found a significant association between practice distance from the hospital, where the MRI would be performed as the intervention, and recruitment with each additional 1 km distance being associated with a 2% reduction in recruitment [77]. Durham et al. investigated the relationship between the distance that potential participants would have to travel between their home address and the clinic [63]. This showed a statistically significant association with a 5% higher participation rate when patients lived in the same zip code as the clinic.

Size of practice: There were inconsistent findings from three studies that investigated practice size—using a proxy of the number of GPs—and recruitment of participants. Fletcher et al. found a strong linear negative association between the number of GPs registered at the practice and the number of patients consenting to take part [47]. Conversely, Brealey et al. found that recruitment was positively associated with the number GPs at the practice [77]. Mclean et al. found no significant difference between the number of GPs in the practice and recruitment rate [71].

##### Research experience 

Powell et al. found no association between the NIHR research level of participating practices and recruitment of participants [51].

##### GP surgery-related barriers 

Dislike of attending the GP practice or practical barriers to attending can negatively influence participation: Attwood et al. found that many interviewees cited barriers with their practice as a reason not to participate [69]. A common theme related to access to the practice, with difficulties relating to appointment systems and reception staff. Other barriers included a perception that their practice was a place to go only when sick, concerns about using up practice time for prevention rather than treatment and a reluctance to receive advice on physical activity when this did not relate to an existing health problem. Normansell et al. also found that a dislike of attending GP surgery was a reason for declining participation [52].

##### Not contactable 

Petty et al. found that several addresses obtained from the practice were incomplete and resulted in some patients not being contacted [60].

#### Practitioner factors

##### Demographic

There is no convincing evidence of an association between the sex of a recruiting practitioner and recruitment. Williams et al. found that females were significantly less likely to recruit than males [75]. This study was at moderate risk of bias. McLean et al. [71] and Richardson et al. [53] found no significant association between sex and recruitment.

##### Clinical experience

McLean et al. found that GPs practising for more than 10 years recruited 4.6% more participants than those practising for less than 10 years [71]. Fletcher et al. also found that GPs who had been qualified the longest (before 1975) recruited the most participants [47]. Williams et al., however, found that years of practice were slightly negatively associated with recruitment [75]. This was, however, no longer statistically significant in multivariable regression analysis and was discounted from the final model.

##### Duration of time at current practice

McLean et al. did not find a significant association between the duration of time that a GP was at their current practice and recruitment rates [71].

##### Working pattern

Richardson et al. found no association between the number of half days worked by the GP and recruitment [53].

##### Professional membership

Williams et al. and Richardson et al. investigated the association with membership status of professional bodies—Royal Australian College of General Practitioners (RACGP) [75] and Pegasus Independent Practitioner Association (IPA) [53]—and found no significant association.

##### Country of training

In a New Zealand-based study, McLean et al. found that GPs trained in New Zealand had a statistically significant, 5% higher, recruitment rate than those trained overseas [71].

##### GP engagement with trial

Williams et al. investigated two measures of GP engagement with a trial on the medical management of low back pain [75]. GPs being readily contactable and routinely screening patients was associated with a significant increase in recruitment.

Durham et al. used whether the eligible participant’s GP was involved in planning the study implementation as a proxy for engagement [63]. They found that there was a statistically significant 5.3% higher participation rate when the GP had been involved in planning.

##### Recruitment study engagement

Richardson et al. found that 56% of GPs who responded to the recruitment study survey after one invitation recruited one or more participants compared with 27.5% of GPs who responded after two or more invites [53]. This was statistically significant.

##### Patient-doctor relationship

A strong patient-doctor relationship can positively influence participation: Van Staa et al. found that seven out of ten participants stated that a *key influencing factor* in their decision to participate in the eLung trial was their excellent relationship with their doctor and trust that it would be in their best interests to participate if their GP asked them [61]. Bleidhorn et al. also found that participants had trusted their GP’s decision in recommending the trial to them [82]. Communication with the GP reassured some patients more than the trial information sheet. The conviction of the GP also influenced the decision of some participants when considering whether they would take part.

However, Petty et al. found that several patients declining participation were concerned that participating in a trial where a pharmacist reviewed their medications could adversely affect their relationship with either their GP or specialist [60].

##### Forgetfulness

Clinicians state that forgetting to recruit is a reason for not including more patients: Van der Gaag et al. found that some GPs simply forgot to recruit participants [48]. One reason given for this was that incident recruitment required having to remember to recruit on an ongoing basis. Another reason was that since there were few eligible patients it was easier to forget. De Blok et al. found that four of ten interviewees mentioned forgetting about the study as a reason for not recruiting [55]. Page et al. showed slight agreement with the 7-point Likert scale statement *During the study period, I forgot to approach patients…* [74]. Forgetting to recruit was also highlighted in the qualitative survey component. Foster et al., however, found that practitioners from both recruiting and non-recruiting groups disagreed with the statement *I forgot to approach patients with asthma to participate* [7].

##### Intention to recruit

Clinicians participating in a trial report having the intention to recruit patients: Foster et al. found that both recruiting and non-recruiting GP groups intended to approach potential participants, with the recruiter group showing a stronger but not significant difference between them [7]. Page et al. also found that GPs intended to invite eligible patients to participate in the study [74].

##### Confusion about the recruitment strategy

One GP participant felt confused regarding the recruitment strategy which impacted on recruitment through failure of implementation [74].

##### Time pressures

Time pressures including competing clinical demands can negatively influence patient recruitment: Interviews by Prout et al. identified many GPs that described time pressures of their surgery impacting their capacity to recruit participants [67]. De Blok et al. found that six of ten GPs state that lack of time was a reason for not recruiting participants [55]. A respondent to Van der Gaag et al. cited many competing organisational demands that affected the capacity to recruit [48]. Page et al. identified one GP who found that the time taken to do the core work of general practice left little time for the trial [74].

##### Not appreciating the importance of the study

De Blok et al. found that five out of ten GPs stated that they did not appreciate the importance of the study [55]. The reasons for this are unclear in the paper.

##### Number of eligible participants

One respondent to Prout et al. felt that there were fewer children attending with URTI than previously and that this limited recruitment [67]. Another respondent to van der Gaag et al*.* cited restrictive eligibility criteria [48].

##### Randomisation concerns

Clinician concern regarding the randomisation of patients can negatively affect patient recruitment: Van der Gaag et al. found that several GPs felt there was a conflict between patients being randomised to a treatment and the normal or preferred paradigm of shared decision-making [48]. Maeland et al. found a similar concern based on the incompatibility of randomisation with shared decision-making [81]. There was also a consistent finding from GPs that randomisation would adversely impact the doctor-patient relationship through the perceived concern of something being left to chance.

Fairhurst et al. interviewed GP participants of a trial that randomised patients to counselling or usual care for minor psychiatric symptoms, to explore reasons for difficulties in recruiting patients [66]. Like Van der Gaag et al., they found that GPs felt that randomisation interfered with decision-making. In this study, they also found that GPs had made their own judgement about what was in the best interests of the patient. If they thought the patient needed counselling, they would generally avoid recruiting them to the trial so that they could offer them counselling independently. Maeland et al. also found that GPs’ own judgement regarding the specific intervention affected their willingness to recruit participants [81]. This again emphasises the importance of clinical equipoise when designing interventions.

##### Concern around delays introduced by the trial

Van der Gaag et al. found that several respondents had concerns regarding the potential delays introduced by entering a participant into the trial [48].

#### Trial factors

##### Inclusion criteria

Durham et al. found a statistically significant, 6.2% increase in the proportion of participants consenting to recruitment when their spouses were also invited to the trial [63]. Rogers et al., however, found no difference in the numbers of participants recruited as a couple or individually [80].

##### Patient recruitment method

Warren et al. compared the time from recruitment of the first participant to the last participant between opportunistic recruitment, where the researcher approached patients in the practice waiting room, and systematic recruitment, where GPs select from a list of potentially eligible participants [50]. The mean time for opportunistic recruitment to be completed was significantly faster at 31.8 days, compared with 86.7 days for systematic recruitment. Markun et al. conducted a non-randomised study of a case-finding approach to identify patients with COPD, for recruitment to a trial [56]. They found that this approach resulted in the additional recruitment of 71 participants which represented 32.9% of the trial participants. Van der Gaag et al. identified incident case recruitment as a main study-related factor affecting poor recruitment [48]. The possible reasons given by GPs for this included the relatively low numbers of cases identified in this way and the requirement for GPs to remember on an ongoing basis. Blair et al. also found that within an incident case recruitment approach, some clinicians were reluctant to recruit less well patients to the study due to the additional time managing their illness would take [54]. They also found that where practices had systems in place to channel patients to higher recruiters, this increased recruitment.

##### Randomisation method

Warren et al. found no association between individual or cluster randomisation of participants and time from recruitment of the first participant to the last participant [50]. Brealey et al. compared postal randomisation and telephone randomisation with the number of participants recruited at each practice. There was a non-significant increase in the number of patients recruited by the telephone randomisation method [77].

##### Financial incentives

Jennings et al. randomised patients to a £100 incentive [49]. This showed a small increase (6.2%) in the number of patients signing a consent form and randomised when offered the incentive. Statistical analysis was only provided for the pooled results of five trials. Four of these were not conducted in general practice; therefore, the pooled results cannot be used in this review.

##### Practice support

Williams et al. investigated two measures of practice support [75]. The first was whether the GP received follow-up by the trial team within 2 weeks from the initial training. This showed a statistically significant association with 2.2 times greater recruitment rate. The second was whether the GP was contacted by the research assistant at least once per month. This did not show a significant association. Richardson et al. found that the assistance of a practice nurse was associated with a significant increase in the numbers of GPs who successfully recruited at least one participant [53].

##### Trial invitation material

Normansell et al. found that the length of time required to read the trial material was a reason for declining, *… there was a lot to read. Bullet points are good. Just make it simple* [52]. Petty et al. identified difficulties in the *readability and comprehension* of the invitation letter, *I had difficulty reading the letter and I had no one to read it for me* [60].

##### Confusion or lack of understanding of the trial

Attwood et al. found that many respondents were unclear about the details of the trial [69]. Two respondents emphasised that some will not read through the participant information in detail. Petty et al. also found that several non-participants had not understood several aspects of the trial, including a misunderstanding of the definition of medicines and a misperception that housebound patients could not be visited at home. Some of this was found to relate to cognitive impairment and deafness.

##### Randomisation concerns

Concerns on being randomised to a specific treatment is a reason for some patients declining participation: Bleidhorn et al. found there were concerns regarding randomisation and not knowing which treatment would be received [82]. Four people who declined had strong preferences for the non-antibiotic treatment arm and this influenced their decision to decline participation [82]. Normansell et al. also found that preferences for specific interventions or to not be in a control group were reasons for declining participation. This raises a conflict in recruiting participants where randomisation creates the robustness of the study but is a barrier to participation due to specific preferences for treatment or concerns due to blinding when present.

##### Intervention unappealing

Lack of interest or dislike of the intervention is a reason for some patients declining participation in trials: Rogers et al. found that for their exercise intervention, there was a reluctance to walk alone, in the evening or in poor weather [80]. There was also a lack of interest in exercise. Concerns were raised regarding the use of an accelerometer due to the perceived discomfort and problems with recording. Normansell et al. also found that a reason for non-participation was dislike of using a pedometer [52]. Walking perceived as boring or during the wrong season was also a reason for declining participation [52].

Normansell et al. found that a barrier was a preference for a group intervention, *I think you get more encouragement if you are in a group* [52].

Rogers et al. identified a mixed preference for one-to-one consultations over group consultations which influenced participation [80].

##### Miscellaneous

Fletcher et al. conducted a time-series analysis that investigated the recruitment rates associated with various changes to the trial design at different time points [57]. The changes investigated included workload reduction for practices, tighter timeframes, a changed approach to practice retention and relaxation of the inclusion criteria. There was a significant increase in recruitment over the last 6 months of the trial; however, since multiple changes were introduced at the same time and due to the critical risk of bias with confounding, the study is unable to determine the cause of changes in recruitment.

De Wit et al. found that the factor *motivation by the participation of the academic research group* survey response predicted the number of patients recruited to the trial [62].

#### Practitioner perception of factors associated with recruitment

Two mixed-methods surveys included items that assessed the perception of practitioners in barriers or facilitators of recruitment for participants [7, 74].

##### Disinterest of potential participants

Foster et al. found there was an agreement with the statement, *I approached patients with asthma to participate but they were not interested* [7]. Page et al. found a slight disagreement with the statement *GP approached patients to participate, but they were not interested* [74]. One of the respondents who felt patients were not interested stated *Few patients were approached and not interested, then I lost the interest*.

##### Patient eligibility

Foster et al. found agreement with the statement *I screened patients with asthma but they were not eligible* [7]. In the same study, there was disagreement with the statement *I did not see any patients who would have been eligible*. Page et al. also found disagreement with the statement *I did not see any patients with acute non-specific low-back pain who would have been eligible for the study* [74].

##### Lack of patient incentives

One of the GP respondents felt that incentives would have helped the recruitment of participants [74].

### Factors associated with the recruitment of practices and practitioners

This section will cover studies that investigate the factors associated with the recruitment of general practices or practitioners as those that will then recruit individual participants. Due to the relative lack of these studies compared to ones focussed on patient recruitment, findings are fewer and of lower confidence.

#### Practice factors

##### Size

The relationship between the size of the practice by a variety of measures and recruitment is unclear from the included studies.

Warren et al. compared the practice list sizes and time to practice the expression of interest [50]. This showed that smaller practices took longer for an expression of interest (EOI) in the trial compared with medium or large practices; however, this difference was not statistically significant. Horspool et al. also found that there was a small positive association between larger practice population size and both the number of practice expressions of interest and the number of practices randomised, although again this association was not statistically significant [65]. Shelton et al. found no difference in the recruitment between GPs working in a solo or group practice [73]. Loskutova et al. examined the relationship between healthcare organisation size [72]. Practices from small organisations were around 3.7 times more likely to participate, but this finding was not statistically significant. Time to recruitment of the organisation from the ‘beginning of recruitment’ until completion of all recruitment paperwork took an average of 71 days. There was no significant difference between the organisation size for this measure. Richardson et al. compared the number of practice respondents to the trial invitation and the number of GPs working in the practice and found that those practices with the most GPs responded the least [53]. This was not statistically significant.

##### Deprivation

Warren et al. found a reduced time to EOI for practices in the least deprived quartile, compared with less deprived quartiles, although this association was not statistically significant [50]. Foster et al. found a significantly higher percentage of non-recruiting GPs (60%) practising in a location of social disadvantage compared with recruiting GPs (53%) [7].

##### Research experience

Horspool et al. found that previous research experience—measured by the number of previous RCTs—had a significantly positive effect on the number of practices randomised to the trial [65]. This is contrary to the study by Powell et al. [51], who found that there was no significant association between practice research experience and the recruitment of participants.

##### Decision made on behalf of practices/practitioners

Loskutova et al. found that decisions to participate in a trial were often made on behalf of potential practice participants by managers [72]. This suggests that clinicians may not be provided with information on the trial to influence trial participation.

##### Ease for the practice

Dormandy et al. interviewed 20 GPs that had participated in a cluster RCT investigating screening in primary care for antenatal sickle cell and thalassaemia to identify their motivations for taking part [59]. One respondent felt motivated by the ease with which their practice could participate.

#### Practitioner factors

##### Age

Richardson et al. found no association between the age categories of GPs and the number of practice respondents to the trial invitation [53]. Shelton et al. found a statistically significant association between age and recruitment to the trial, with participants being an average of 4.3 years younger than non-participants [73].

##### Sex

Shelton et al. found a higher number of participants were male; however, a higher proportion of women participated (85.7%), compared with men (67%). This was not statistically significant [73].

##### Rurality

Shelton et al. found that a greater number of participating practitioners were located in an urban area compared to rural practitioners [73]. However, a higher proportion of rural practitioners participated than declined. This was statistically significant.

##### Readiness to change

Shelton et al. measured the practitioners’ readiness to change cancer screening and counselling behaviours—the focus of the intervention—and found no significant difference between participating and declining physicians [73].

##### Altruism

Brodaty et al. found that the survey item *altruism/desire to contribute to research* was one of the most common motivating factors clinicians gave for participating [76]. Ellis et al. found that relatively few GPs were motivated to participate due to a *desire to help my colleagues* [78].

##### Collaboration

Brodaty et al. found that more non-participants agreed with the survey item *Collaborate with other professionals/form or strengthen contacts* as a motivating factor for potential participation [76]. Five (50%) non-participants provided this as a reason compared with 3 (30%) in the intervention group and 2 (20%) in the control group. Gunn et al. found that collaboration was important to GPs with 22 (75%), responding very or quite for the degree to which they agreed with the survey item *I enjoyed collaborating with other professionals (both GPs and non-GPs)* [79]*.*

##### Continuing medical education (CME) points

Provision of CME points is an important positive influencing factor for many GPs: Brodaty et al. found similar importance between intervention, control and refuser groups in the number of responses to the survey item ‘Fulfil CME requirements’ [76] with around half agreeing from all groups. Gunn et al. found a similar importance to the survey item *it enabled me to fulfil my CME requirements*, with 12 (42%) respondents agreeing that the degree to which it influenced their decision was very or quite [79]. Ellis et al. surveyed GPs and found the *ability to earn convenient CME credit* was provided as a reason for participating in the trial by 37 respondents (19%) [78]. Pearl et al. found that 23 (38%) GPs agreed or strongly agreed that Maintenance of Professional Standards (MOPS) points were an important part of their decision to take part.

##### Doctor-patient relationship

Brodaty et al. found that 9 (30%) of GPs were motivated to participate in the trial to *improve* [the] *doctor-patient relationship* [76].

##### Personal relationship with the researcher

Rarely did any respondents to Brodaty et al. agree that a personal relationship with the researcher was a motivating factor for considering participation in the trial [76].

##### Helping patients

The potential for clinicians to help patients is an important factor positively influencing participating: Brodaty et al. found the survey item *help my patients further* received a high number of responses with 13 (65%) participating GPs and 8 (80%) of declining GPs providing this as a motivating factor in considering participation [76].

Gunn et al. also found this was one of the most important reasons given for GPs to participate in their trial [79]. To the survey item *it helped my own patients*, 27 (93%) responded very or quite for the degree that this influenced their decision. The item *in time it will help patients elsewhere*, was important but less than helping the GPs’ own patients, with 24 (82%) answering very or quite [79].

##### Reflecting on and improving practice

Clinicians are motivated to participate when the research can improve clinical practice: Brodaty et al. found that a large minority of GPs agreed that a motivating factor for considering participation was reflecting on their practice [76]. Gunn et al. found that it was more important to their clinicians with 26 (89%), responding very or quite for the survey item *it allowed me to reflect on the way I practise* for the degree to which it influenced their decision [79]. Ellis et al. found that the item *Interested in improving my clinical practice* was the most commonly given response for why GPs participated with 157 (81%) responding to this [78]. Brodaty et al. found that the most common reason given as a motivating factor to consider participation in the trial was to update knowledge [76]. Gunn et al. also found that a major reason for 27 (93%) GPs participating in the trial was to update their knowledge and to learn new clinical skills [79]. This was supported by answers to the open-ended questions where *most of the GPs reported their desire to gain knowledge and clinical skills*.

##### Research interest and perception of research value

An interest in ‘research’ itself was generally not important in influencing participation: Loskutova et al. interviewed managers who often acted as *gatekeeper* to decision-makers of the healthcare organisation [72]. They found an opinion that despite the busyness of the clinicians, a *special interest* in the research topic could increase motivation to participate. Brodaty et al. found that the survey items *Interest in the research question/area* and *Learn more about research* were relatively unimportant to GP respondents as a motivating factor [76]*.* In response to the survey item *I provided the research team with knowledge and expertise from the ‘real world’ of general practice*, Gunn et al. found that 18 (62%) answered very or quite for the degree to which this influenced their decision [79]. The general theme that GPs were not especially interested in research is supported by free-text answers where *only five GPs mentioned their desire to contribute to or learn about research*. Ellis et al. received 61 (31%) responses stating that a motivation for participating in the trial was because they *like to remain involved in research initiatives* [78]. A total of 106 (54%) respondents felt that a *specific* interest in *…contributing to primary prevention for coronary vascular disease* was a motivating factor. Prout et al. found that several GPs wanted to participate due to the *perceived intrinsic value of research* [67]. Following interviews with GPs declining trial participation, Salmon et al. concluded that *in a hierarchy of actual or potential activities for these GPs, research was low in its clinical or professional value* [64].

##### Time constraints

Demands on clinicians’ time and the trials’ impact on this influences decisions to participate: Loskutova et al. found that the high workload of health care organisations impacted the likelihood of participation in their trial, *I have four current projects I am working on … and I just do not feel I can commit to another right now* [72]. Brodaty et al. also found in interviews with GPs that lack of time was cited as a barrier to participation in the trial [76]. On interviewing declining GPs, Shelton et al. found that *being too busy* was given as a reason for declining by 9 of 13 (69%) GPs. Van Staa et al*.* found that *nearly all GPs in this sample described … significant increases in workload, reporting requirements and patient demand*. The GPs suggested that deciding to participate in a trial with respect to time would require the trial to fit well with the existing workflow. Low perceived time pressures of the SAVIT study were found to be a positive aspect with one GP respondent to Prout et al., stating that other studies in which they had participated were not feasible due to time requirements [67]. Salmon et al. found that *the lack of time for research characterized all GPs’ accounts* [64].

#### Trial factors

##### Clinical relevance

Perceived clinical relevance of the trial is an important influence on deciding to participate: Van Staa et al. found that *Most GPs also identified the need for the research to be locally relevant and clinically important for the local population* [61]. The potential benefit to the local population was stated as being a positive influencing factor for participating in the eLung trial by 13 (48.1%) GPs and was the most important factor in participating for four (14.8%) GPs. In the same study, alignment of the eLung protocol with existing local prescribing guidance on the management of COPD exacerbations was the most commonly stated positive influencing factor when deciding on participation. Prout et al. found that clinicians were concerned regarding the lack of treatment for URTI, and the trial was thought to potentially improve this [67]. Dormandy et al. found a major theme motivating GP participation was that of clinical importance [59]. Brodaty et al. found in interview that GPs preferred *research relevant to their role at the ‘coal face’ of dementia services* [76].

##### Practice recruitment method

Colwell et al. investigated a viral marketing approach to practice recruitment in a non-randomised study of an intervention [58]. In Sheffield, where this marketing approach was implemented, a greater number of practices consented compared to Doncaster and Rotherham, where usual recruitment practices were followed. This was scored at critical risk of bias due to confounding. Ellis et al. also conducted a retrospective analysis of ten recruitment strategies used when recruiting [78]. These were classified as opt-in, opt-out and a combination of both strategies. The success rate of these recruitment strategies, defined by the number of practices recruited to the trial over the number of *practitioners* exposed to the recruitment strategy, ranged from 0 to 42%. As with Colwell et al. this study was also a NRSI and was scored as being at critical risk of bias.

##### Participant recruitment method

Van Staa et al. found that recruitment during a routine consultation was a controversial component of the eLung trial due to time pressures [61]. Six (50%) GPs declining participation cited this as a negative influencing factor. Opportunistic recruitment was a positive influencing factor for 6 (22.2%) GPs due to *the need for the recruitment method to be as simple and efficient as possible…*.

##### Perceived ease of recruiting patients

**Computer-based pop-up alerts** Van Staa et al. found that the use of pop-up alerts to identify, screen and recruit participants was the fourth most influential factor for 10 (37%) GPs that identified this as a positive factor, and 3 (11%) of GPs who identified it as a negative factor [61]. The negative views related to dislike of pop-ups within current GP systems and a wish to avoid additional alert burden. GPs who felt these were a positive factor identified their potential to save time and reduce workload.

**Number of eligible patients** Van Staa et al. also found that the feasibility of recruitment was considered a positive influencing factor by 13 GPs (48.1%) and a negative influencing factor by 10 GPs (37.0%) and was the most commonly mentioned negative factor influencing GPs’ decision on participation [61]. Concerns related to a low incidence of COPD and the impact of pre-existing rescue packs (one of the interventions) on eligible numbers. Those GPs that considered the feasibility of recruitment as a positive factor thought there would be a large number of eligible patients and minimal reduction in numbers caused by pre-existing use of rescue packs.

##### Incentives

**Financial incentives** Financial incentives can be an important positive influencing factor in decisions to participate but are seldom the most important: Loskutova et al. identified through interviews with healthcare organisations that clinicians may participate if *enough money* was provided to generate interest [72]. Brodaty et al. found that GPs seldom reported financial reimbursement as being a motivating factor in deciding on participation [76]. Five (25%) GP participants and 1 (10%) GP refuser agreed with the survey item *Receive Medicare payments for my patients’ 75+ health assessments at $171 or $200 per assessment*. This was supported by interviews where the authors state that *they appreciated remuneration but did not rank this highly.* De Wit et al. also found that financial incentives were important in the initial motivation for participation in a minority of GPs only (no further data since tables are not available) [62]. Van Staa et al. found that for 13 (48.1%) GPs, financial remuneration was a positive influencing factor [61]. Three (11.1%) GPs identified making a profit as the most important factor when deciding and covering study costs was the most important to one (3.7%). Salmon et al. found in interview that reimbursement could persuade GPs to participate by giving up their own time [64]. Payment was also reported as a way of increasing interest in the trial.

**Other incentives** Incentives such as equipment provision, *wanted to receive PDA* and *wanted to receive automated blood pressure device*, were uncommon motivators for participation [78].

##### Miscellaneous

**Professional endorsement** Brodaty et al. found that GPs rarely reported that professional endorsement by the RACGP or their division was a motivating factor in deciding on participating in the trial [76].

**Training sessions** Prout et al. found that *initial training sessions were well liked since this allowed the clinicians an opportunity to discuss the trial prior to agreeing to participate* [67].

## Discussion

This systematic review investigated patient, practice, practitioner and trial factors associated with the recruitment of patients, practices and practitioners to RCTs in general practice.

Many studies have been conducted to investigate the effect of interventions or association of factors and recruitment. The majority of these have been ad hoc retrospective studies which have been at high risk of bias or designed in such a way that they cannot provide sufficient evidence to determine what improves recruitment and what does not. This finding of poor-quality recruitment studies has been noted by other researchers [12, 15]. For these reasons, it is difficult to make firm recommendations to trialists. However, the following are factors that were identified during the review as a deserving particular focus. These tended to come from qualitative studies or surveys.

For practitioners and patients alike, a trial that is clinically relevant is critical in influencing participation. Similarly, Caldwell et al. found that *recruitment strategies that focus on increasing potential participants’ awareness of the health problem being studied* [and] *its potential impact on their health appeared to increase recruitment to clinical studies* [86]. In an overview of systematic reviews of psychosocial barriers and facilitators to recruitment, Sheridan et al. also found that personal benefit for potential patient participants was a facilitator to recruitment in 20 systematic reviews [87]. Clinical equipoise is important in a trial since clinicians will understandably act in the perceived best interests of the *current* patient, over generating new knowledge to help *future* patients. Competing demands are given as an important reason for declining participation. In a Cochrane systematic review, Houghton et al. also found that perceived time commitment was a concern for potential patient participants [14]. Sahin et al. found 11 studies citing clinicians perceived lack of time as a reason against the recruitment of patients [88]. The pressures on frontline staff are also increasing which exacerbates this issue [89]. For patients and clinicians, there are concerns about randomisation relating to its impact on shared decision-making and not knowing which treatment will be assigned. Sheridan et al. found seven systematic reviews that reported patient aversion to individual patient randomisation as a barrier to participation [87]. A Zelen or patient preference design may be considered as a way of addressing this issue [90, 91].

Patients make decisions about whether they are a ‘candidate’ for the trial even when they are objectively eligible. This is a new finding and should be explored further. General practice processes can hinder recruitment and a strong pre-existing doctor-patient relationship can improve recruitment. Houghton et al. found that potential patient participants *place huge trust* in healthcare professionals who therefore have *great potential* to influence participation [14].

For clinicians, the wish to help research itself is seldom an important reason for participating. Contrary to this, Houghton et al. found that for some potential patient participants, there was *genuine curiosity and interest in contributing to the trial and scientific knowledge* [14]. This difference between patient and clinician motivation is important to consider.

One of the few experimental findings was that opportunistic recruitment resulted in significantly faster recruitment compared to systematic recruitment.

Methodologically, recruitment research of practices and practitioners should have increased priority. Higher quality, including experimental studies of recruitment, are required to find out what works rather than what might work. This finding is aligned with the Trial Forge initiative which aims to improve the evidence base for conducting trials, including through the use of Studies Within A Trial (SWATs )[92].

Factors influencing recruitment have clear implications for trial design and will be used by the authors in specifying potential predictor variables for inclusion in a predictive model of recruitment to a range of future trials.

### Strengths

To the best of our knowledge, this is the first systematic review that has investigated recruitment to RCTs, specifically in general practice, and that has included the recruitment of patients, practitioners and practices. This was underpinned by a rigorous systematic review methodology, including search strategy, screening, analysis and synthesis.

### Limitations

Studies were generally of poor quality which limited the number of findings that could be made. Studies were also highly heterogeneous both in dependent and independent variables. This prevented a meta-analysis for the quantitative studies which may have led to clearer results for those studies. Information gaps in the studies are evident in the predilection for studies of patient participants, rather than practice and practitioner participants. This limited the useful information that could be gained in these areas. Studies were of English language only; however, no non-English studies were excluded at the final screening stage. The synthesis section was carried out independently by one researcher (KM); however, the themes and findings generated were reviewed by another experienced researcher (FS).

This review has focused on recruitment in a broad sense. Issues such as heterogeneity within the trial population were not within the scope of this review. It should be noted, however, that trials in general practice have the added complexity of heterogeneity of both the practice and patient population.

### Suggestions for further research

A similar conclusion to our review was reached by Gardner et al. (70), who conducted a systematic review investigating non-randomised interventions to improve participant recruitment to RCTs. Studies should also increase their focus on the recruitment of practice and practitioners since there was a clear predilection for studies of patient recruitment. Also, while it would be more difficult to find out more about non-participants this is likely to be a helpful addition to the focus on those that have participated. Studying participants provides insights into why people participate, we also need to know why people did not.

The concept of candidacy and its impact on recruitment needs further investigation, both to confirm that this has value in explaining recruitment and to explore interventions to improve candidacy. This could include actively addressing this through trial information and discussions to make clear that any patient who fulfils the eligibility criteria is ‘the right person for this trial’.

Richardson et al. found that engagement with the recruitment study itself was associated with recruitment to the RCT [53]. There is low confidence in this due to the high risk of bias with that study. However, this factor provides a useful hypothesis for further research. For example, do initial response rates to the first invitation to an RCT reliably predict engagement with the trial throughout?

## Conclusion

This review has identified many factors associated with the recruitment of patients, practices and practitioners to RCTs. Clinical relevance, competing demands, randomisation concerns, patients perception of ‘candidacy’, general practice processes, doctor-patient relationship and recruitment method are all important in influencing recruitment.

Methodologically, recruitment research of practices and practitioners should have increased priority. Higher quality studies of recruitment are urgently required to find out what actually works rather than what might work.

## Supplementary Information

**Additional file 1.** PRISMA 2020 Checklist.

**Additional file 2.** Search strategies.

**Additional file 3.** Summary of study characteristics.

**Additional file 4.** Assessment of methodological limitations.

**Additional file 5.** Quality appraisal summary.

**Additional file 6.** Quantitative results tabulation.

**Additional file 7.** Qualitative results tabulation.

**Additional file 8.** Glossary.

## Data Availability

Screening forms, template data collection forms, data extracted from included studies and nVIVO analysis file are available at the following link https://osf.io/vjhk7/?view_only=142ea515cc3c475798b98d0e8580c674.

## References

[1] Walters SJ, dos Anjos B, Henriques-Cadby I, Bortolami O, Flight L, Hind D, et al. Recruitment and retention of participants in randomised controlled trials: a review of trials funded and published by the United Kingdom Health Technology Assessment Programme. BMJ Open. 2017;7(3). 10.1136/bmjopen-2016-015276. 10.1136/bmjopen-2016-015276 PMC5372123 28320800

[2] McDonald AM, Knight RC, Campbell MK, Entwistle VA, Grant AM, Cook JA, Elbourne DR, Francis D, Garcia J, Roberts I (2006). What influences recruitment to randomised controlled trials? A review of trials funded by two UK funding agencies. Trials.

[3] Sully BG, Julious SA, Nicholl J (2013). A reinvestigation of recruitment to randomised, controlled, multicenter trials: a review of trials funded by two UK funding agencies. Trials.

[4] Bower P, Wilson S, Mathers N (2007). Short report: how often do UK primary care trials face recruitment delays?. Fam Pract.

[5] van der Gaag WH, van den Berg R, Koes BW, Bohnen AM, Hazen LM, Peul WC, et al. Discontinuation of a randomised controlled trial in general practice due to unsuccessful patient recruitment. BJGP Open. 2017;1(3). 10.3399/bjgpopen17X101085. 10.3399/bjgpopen17X101085 PMC6169930 30564680

[6] Schreijenberg M, Luijsterburg PAJ, Van Trier YDM, Rizopoulos D, Koopmanschap MA, Voogt L, Maher CG, Koes BW (2018). Discontinuation of the PACE Plus trial: problems in patient recruitment in general practice. BMC Musculoskelet Disord.

[7] Foster JM, Sawyer SM, Smith L, Reddel HK, Usherwood T (2015). Barriers and facilitators to patient recruitment to a cluster randomized controlled trial in primary care: lessons for future trials. BMC Med Res Methodol.

[8] Tudur Smith C, Hickey H, Clarke M, Blazeby J, Williamson P (2014). The trials methodological research agenda: results from a priority setting exercise. Trials.

[9] Healy P, Galvin S, Williamson PR, Treweek S, Whiting C, Maeso B, Bray C, Brocklehurst P, Moloney MC, Douiri A (2018). Identifying trial recruitment uncertainties using a James Lind Alliance Priority Setting Partnership – the PRioRiTy (Prioritising Recruitment in Randomised Trials) study. Trials.

[10] Treweek S, Pitkethly M, Cook J, Kjeldstrom M, Taskila T, Johansen M, et al. Strategies to improve recruitment to randomised controlled trials. Cochrane Database Syst Rev. 2010;4. 10.1002/14651858.MR000013.pub5. 10.1002/14651858.MR000013.pub5 20393971

[11] Treweek S, Lockhart P, Pitkethly M, Cook JA, Kjeldstrom M, Johansen M, et al. Methods to improve recruitment to randomised controlled trials: Cochrane systematic review and meta-analysis. BMJ Open. 2013;3(2). 10.1136/bmjopen-2012-002360. 10.1136/bmjopen-2012-002360 PMC3586125 23396504

[12] Treweek S, Pitkethly M, Cook J, Fraser C, Mitchell E, Sullivan F, Jackson C, Taskila TK, Gardner H (2018). Strategies to improve recruitment to randomised trials. Cochrane Database Syst Rev.

[13] Bell-Syer SE, Thorpe LN, Thomas K, Macpherson H (2011). GP participation and recruitment of patients to RCTs: lessons from trials of acupuncture and exercise for low back pain in primary care. Evid Based Complement Alternat Med.

[14] Houghton C, Dowling M, Meskell P, Hunter A, Gardner H, Conway A, et al. Factors that impact on recruitment to randomised trials in health care: a qualitative evidence synthesis. Cochrane Database Syst Rev. 2020;10. 10.1002/14651858.MR000045.pub2. 10.1002/14651858.MR000045.pub2 PMC8078544 33026107

[15] Gardner H, Albarquoni L, El Feky A, Gillies K, Treweek S. A systematic review of non-randomised evaluations of strategies to improve participant recruitment to randomised controlled trials. F1000Res. 2020;9(86). 10.12688/f1000research.22182.1. 10.12688/f1000research.22182.1 PMC7336048 32685133

[16] Primary Care: Scottish Government; 2018. Available from: http://www.gov.scot/Topics/Health/Services/Primary-Care.

[17] The world health report 2008 : primary health care : now more than ever. Geneva: World Health Organisation (WHO); 2008.

[18] Hone T, Macinko J, Millett C (2018). Revisiting Alma-Ata: what is the role of primary health care in achieving the Sustainable Development Goals?. Lancet.

[19] Sullivan F, Hinds A, Pitkethly M, Treweek S, Wilson P, Wyke S (2014). Primary care research network progress in Scotland. Eur J Gen Pract.

[20] Rørtveit G (2014). Research networks in primary care: an answer to the call for better clinical research. Scand J Prim Health Care.

[21] Mold JW (2012). Primary care research conducted in networks: getting down to business. J Am Board Fam Med.

[22] Barnard KD, Dent L, Cook A (2010). A systematic review of models to predict recruitment to multicentre clinical trials. BMC Med Res Methodol.

[23] Gkioni E, Rius R, Dodd S, Gamble C (2019). A systematic review describes models for recruitment prediction at the design stage of a clinical trial. J Clin Epidemiol.

[24] Heitjan DF, Ge Z, Ying GS (2015). Real-time prediction of clinical trial enrollment and event counts: a review. Contemp Clin Trials.

[25] Anisimov VV. Modern analytic techniques for predictive modeling of clinical trial operations. In: Quantitative methods in pharmaceutical research and development: Switzerland: Springer; 2020. p. 361–408.

[26] Liu J, Allen PJ, Benz L, Blickstein D, Okidi E, Shi X (2021). A machine learning approach for recruitment prediction in clinical trial design. arXiv preprint arXiv:211107407.

[27] Page MJ, McKenzie JE, Bossuyt PM, Boutron I, Hoffmann TC, Mulrow CD, Shamseer L, Tetzlaff JM, Akl EA, Brennan SE (2021). The PRISMA 2020 statement: an updated guideline for reporting systematic reviews. Bmj.

[28] PROSPERO International prospective register of systematic reviews [09/10/2021]. Available from: https://www.crd.york.ac.uk/prospero/.

[29] Moffat KR, Cannon P, Shi W, Sullivan F (2019). Factors associated with recruitment to randomised controlled trials in general practice: protocol for a systematic review. Trials.

[30] Rethlefsen ML, Kirtley S, Waffenschmidt S, Ayala AP, Moher D, Page MJ, Koffel JB, Blunt H, Brigham T, Chang S (2021). PRISMA-S: an extension to the PRISMA Statement for Reporting Literature Searches in Systematic Reviews. Syst Rev.

[31] OpenGrey [27/09/2021]. Available from: http://www.opengrey.eu/.

[32] National Technical Reports Library 2014 [27/09/2021]. Available from: https://ntrl.ntis.gov/NTRL/.

[33] BMC Trials: Springer Nature; 2021 [04/10/21]. Available from: https://trialsjournal.biomedcentral.com/.

[34] Booth A, Sutton A, D P: Systematic pproaches to a successful literature review, 2nd SAGE Publishing; 2016.

[35] PubMed 2021 [04/10/21]. Available from: https://pubmed.ncbi.nlm.nih.gov/.

[36] Cooke A, Smith D, Booth A (2012). Beyond PICO: the SPIDER tool for qualitative evidence synthesis. Qual Health Res.

[37] Sterne JAC, Savović J, Page MJ, Elbers RG, Blencowe NS, Boutron I, Cates CJ, Cheng H-Y, Corbett MS, Eldridge SM (2019). RoB 2: a revised tool for assessing risk of bias in randomised trials. Bmj.

[38] Downes MJ, Brennan ML, Williams HC, Dean RS (2016). Development of a critical appraisal tool to assess the quality of cross-sectional studies (AXIS). BMJ Open.

[39] CASP Checklists: Critical Appraisal Skills Programme; 2018 [26/06/2018]. Available from: https://casp-uk.net/casp-tools-checklists/.

[40] Sterne JA, Hernán MA, Reeves BC, Savović J, Berkman ND, Viswanathan M, Henry D, Altman DG, Ansari MT, Boutron I (2016). ROBINS-I: a tool for assessing risk of bias in non-randomised studies of interventions. Bmj.

[41] Pluye P, Gagnon MP, Griffiths F, Johnson-Lafleur J (2009). A scoring system for appraising mixed methods research, and concomitantly appraising qualitative, quantitative and mixed methods primary studies in mixed studies reviews. Int J Nurs Stud.

[42] Current version of RoB 2 [28/09/2021]. Available from: https://sites.google.com/site/riskofbiastool/welcome/rob-2-0-tool/current-version-of-rob-2.

[43] ROBINS-I detailed guidance. 2016 [28/09/2021]. Available from: https://sites.google.com/site/riskofbiastool/welcome/home/current-version-of-robins-i/robins-i-detailed-guidance-2016.

[44] University of York. Centre for Reviews and Dissemination. Systematic reviews: CRD’s guidance for undertakingreviews in health care. York: Centre for Reviews and Dissemination; 2009. Available from:http://www.york.ac.uk/inst/crd/SysRev/!SSL!/WebHelp/SysRev3.htm.

[45] Thomas J, Harden A (2008). Methods for the thematic synthesis of qualitative research in systematic reviews. BMC Med Res Methodol.

[46] Hong QN, Pluye P, Bujold M, Wassef M (2017). Convergent and sequential synthesis designs: implications for conducting and reporting systematic reviews of qualitative and quantitative evidence. Syst Rev.

[47] Fletcher K, Mant J, Holder R, Fitzmaurice D, Lip GYH, Hobbs FDR (2007). An analysis of factors that predict patient consent to take part in a randomized controlled trial. Fam Pract.

[48] van der Gaag WH, van den Berg R, Koes BW, Bohnen AM, Hazen LMG, Peul WC, et al. Luijsterburg PAJ: Discontinuation of a randomised controlled trial in general practice due to unsuccessful patient recruitment. BJGP Open. 2017;1(3). 10.3399/bjgpopen17X101085. 10.3399/bjgpopen17X101085 PMC6169930 30564680

[49] Claudine G, Jennings TMM, Li Wei MJB, Mcconnachie L, Mackenzie IS. Does offering an incentive payment improve recruitment to clinical trials and increase the proportion of socially deprived and elderly participants? BMC Trials. 2015;16(1):80. 10.1186/s13063-015-0582-8 PMC4364332 25888477

[50] Warren FC, Stych K, Thorogood M, Sharp DJ, Murphy M, Turner KM, Holt TA, Searle A, Bryant S, Huxley C (2014). Evaluation of different recruitment and randomisation methods in a trial of general practitioner-led interventions to increase physical activity: a randomised controlled feasibility study with factorial design. Trials [Electronic Resource].

[51] Powell K, Wilson VJ, Redmond NM, Gaunt DM, Ridd MJ (2016). Exceeding the recruitment target in a primary care paediatric trial: an evaluation of the Choice of Moisturiser for Eczema Treatment (COMET) feasibility randomised controlled trial. Trials [Electronic Resource].

[52] Normansell R, Holmes R, Victor C, Cook DG, Kerry S, Iliffe S, Ussher M, Fox-Rushby J, Whincup P, Harris T (2016). Exploring non-participation in primary care physical activity interventions: PACE-UP trial interview findings. Trials [Electronic Resource].

[53] Richardson A, Sutherland M, Wells E, Toop L, Plumridge L (2002). Factors affecting general practitioner involvement in a randomised controlled trial in primary care. N Z Med J.

[54] Blair PS, Turnbull S, Ingram J, Redmond N, Lucas PJ, Cabral C, Hollinghurst S, Dixon P, Peters T, Horwood J (2017). Feasibility cluster randomised controlled trial of a within-consultation intervention to reduce antibiotic prescribing for children presenting to primary care with acute respiratory tract infection and cough. BMJ Open.

[55] Flokstra-de Blok BMJ, Brakel TM, Wubs M, Skidmore B, Kocks JWH, Oude Elberink JNG, Schuttelaar MA, van der Velde JL, van der Molen T, Dubois AEJ (2018). The feasibility of an allergy management support system (AMSS) for IgE-mediated allergy in primary care. Clin Transl Allergy.

[56] Markun S, Rosemann T, Dalla-Lana K, Steurer-Stey C (2016). The impact of case finding on the recruitment yield for COPD research in primary care: an observational study. Respiration.

[57] Fletcher K, Mant J, Roalfe A, Hobbs FDR (2010). Impact of study design on recruitment of patients to a primary care trial: an observational time series analysis of the Birmingham Atrial Fibrillation Treatment of the Aged (BAFTA) Study. Fam Pract.

[58] Colwell B, Mathers N, Ng CJ, Bradley A (2012). Improving recruitment to primary care trials: some lessons from the use of modern marketing techniques. Br J Gen Pract.

[59] Dormandy E, Kavalier F, Logan J, Harris H, Ishmael N, Marteau TM, Anionwu EN, Atkin K, Brown K, Bryan S (2008). Maximising recruitment and retention of general practices in clinical trials: a case study. Br J Gen Pract.

[60] Petty DR, Zermansky AG, Raynor DK, Vail A, Lowe CJ, Freemantle N, Buttress AD (2001). “No thank you”: why elderly patients declined to participate in a research study. Pharm World Sci.

[61] Staa TP, Dyson L, McCann G, Padmanabhan S, Belatri R, Goldacre B, Cassell J, Pirmohamed M, Torgerson D, Ronaldson S (2014). The opportunities and challenges of pragmatic point-of-care randomised trials using routinely collected electronic records: evaluations of two exemplar trials. Health Technol Assess (Winch Eng).

[62] Wit NJ, Quartero AO, Zuithoff AP, Numans ME (2001). Participation and successful patient recruitment in primary care. J Fam Pract.

[63] Durham ML, Beresford S, Diehr P, Grembowski D, Hecht JA, Patrick DL (1991). Participation of higher users in a randomized trial of Medicare reimbursement for preventive services. Gerontologist.

[64] Salmon P, Peters S, Rogers A, Gask L, Clifford R, Iredale W, Dowrick C, Morriss R (2007). Peering through the barriers in GPs’ explanations for declining to participate in research: the role of professional autonomy and the economy of time. Fam Pract.

[65] Horspool MJ, Julious SA, Mooney C, May R, Sully B, Smithson WH (2015). Preventing and Lessening Exacerbations of Asthma in School-aged children Associated with a New Term (PLEASANT): recruiting primary care research sites-the PLEASANT experience. NPJ Prim Care Respir Med.

[66] Fairhurst K, Dowrick C (1996). Problems with recruitment in a randomized controlled trial of counselling in general practice: causes and implications. J Health Serv Res Policy.

[67] Prout H, Butler C, Kinnersley P, Robling M, Hood K, Tudor-Jones R (2003). A qualitative evaluation of implementing a randomized controlled trial in general practice. Fam Pract.

[68] Pearl A, Wright S, Gamble G, Doughty R, Sharpe N. Randomised trials in general practice - a New Zealand experience in recruitment. N Z Med J. 2003;116(1186):U681. 14657964

[69] Attwood S, Morton KL, Mitchell J, Emmenis MV, Sutton S, Team VBIP (2016). Reasons for non-participation in a primary care-based physical activity trial: a qualitative study. BMJ Open.

[70] Welsh JL, Adam P, Fontaine P, Gjerdingen D (2002). Recruiting for a randomized controlled trial from an ethnically diverse population: lessons from the Maternal Infection and Preterm Labor Study. J.

[71] McLean C, Kerse N, Moyes SA, Ng T, Lin SYS, Peri K (2014). Recruiting older people for research through general practice: the Brief Risk Identification Geriatric Health Tool trial. Australas J Ageing.

[72] Loskutova NY, Smail C, Ajayi K, Pace WD, Fox CH (2018). Recruiting primary care practices for practice-based research: a case study of a group-randomized study (TRANSLATE CKD) recruitment process. Fam Pract.

[73] Shelton BJ, Wofford JL, Gosselink CA, McClatchey MW, Brekke K, Conry C, Wolfe P, Cohen SJ (2002). Recruitment and retention of physicians for primary care research. J Community Health.

[74] Page MJ, French SD, McKenzie JE, Connor DAO, Green SE (2011). Recruitment difficulties in a primary care cluster randomised trial: investigating factors contributing to general practitioners’ recruitment of patients. BMC Med Res Methodol.

[75] Williams CM, Maher CG, Hancock MJ, McAuley JH, Lin CWC, Latimer J (2014). Recruitment rate for a clinical trial was associated with particular operational procedures and clinician characteristics. J Clin Epidemiol.

[76] Brodaty H, Gibson LH, Waine ML, Shell AM, Lilian R, Pond CD (2013). Research in general practice: a survey of incentives and disincentives for research participation. Ment Health Fam Med.

[77] Brealey SD, Atwell C, Bryan S, Coulton S, Cox H, Cross B, Fylan F, Garratt A, Gilbert FJ, Gillan MG (2007). Using postal randomization to replace telephone randomization had no significant effect on recruitment of patients. J Clin Epidemiol.

[78] Ellis SD, Bertoni AG, Bonds DE, Clinch CR, Balasubramanyam A, Blackwell C, Chen H, Lischke M, Goff DC (2007). Value of recruitment strategies used in a primary care practice-based trial. Contemp Clin Trials.

[79] Gunn J, McCallum Z, Sanci L (2008). What do GPs get out of participating in research? - experience of the LEAP trial. Aust Fam Physician.

[80] Rogers A, Harris T, Victor C, Woodcock A, Limb E, Kerry S, Iliffe S, Whincup P, Ekelund U, Beighton C (2014). Which older people decline participation in a primary care trial of physical activity and why: insights from a mixed methods approach. BMC Geriatr.

[81] Maeland S, Magnussen LH, Eriksen HR, Malterud K (2011). Why are general practitioners reluctant to enrol patients into a RCT on sick leave? A qualitative study. Scand J Public Health.

[82] Bleidorn J, Bucak S, Gagyor I, Hummers-Pradier E, Dierks ML (2015). Why do - or don’t - patients with urinary tract infection participate in a clinical trial? A qualitative study in German family medicine. German. Med Sci.

[83] Davison C, Smith GD, Frankel S (1991). Lay epidemiology and the prevention paradox: the implications of coronary candidacy for health education. Sociol Health Illn.

[84] Hunt K, Emslie C (2001). Commentary: the prevention paradox in lay epidemiology--Rose revisited. Int J Epidemiol.

[85] Tookey S, Renzi C, Waller J, von Wagner C, Whitaker KL (2018). Using the candidacy framework to understand how doctor-patient interactions influence perceived eligibility to seek help for cancer alarm symptoms: a qualitative interview study. BMC Health Serv Res.

[86] Caldwell PHY, Hamilton S, Tan A, Craig JC (2010). Strategies for increasing recruitment to randomised controlled trials: systematic review. PLoS Med.

[87] Sheridan R, Martin-Kerry J, Hudson J, Parker A, Bower P, Knapp P (2020). Why do patients take part in research? An overview of systematic reviews of psychosocial barriers and facilitators. Trials.

[88] Sahin D, Yaffe MJ, Sussman T, McCusker J (2014). A mixed studies literature review of family physicians’ participation in research. Fam Med.

[89] Peckham S, Eida T, Zhang W, Hashem F, Spencer S, Kendall S, Newberry Le Vay J, Buckley-Mellor O, Samuel E, Vohra J (2021). Creating time for research: identifying and improving the capacity of healthcare staff to conduct research. Cancer Research UK.

[90] Bower P, King M, Nazareth I, Lampe F, Sibbald B (2005). Patient preferences in randomised controlled trials: conceptual framework and implications for research. Soc Sci Med.

[91] Torgerson DJ, Roland M (1998). What is Zelen’s design?. Bmj.

[92] Treweek S, Bevan S, Bower P, Campbell M, Christie J, Clarke M, Collett C, Cotton S, Devane D, El Feky A (2018). Trial forge guidance 1: what is a Study Within A Trial (SWAT)?. Trials.

