# Supplementary material for: Factors associated with recruitment to randomised controlled trials in general practice: a systematic mixed studies review
Source: Trials. 2023 Feb 6;24:90. doi: 10.1186/s13063-022-06865-x (PMC9903494; doi:10.1186/s13063-022-06865-x)
Supplement: Supplementary file 2 — Additional file 2. Search strategies. [file 13063_2022_6865_MOESM2_ESM.docx]

**Original Search - 23 May 2018**

**Source: Ovid MEDLINE(R) In-Process & Other Non-Indexed Citations and Ovid MEDLINE**

Interface: OvidSP

Database coverage dates: 1946 to Present

Search date: 23 May 2018

Retrieved records: 2565

Search strategy:

1 exp Clinical Trials as Topic/ (314264)

2 Multicenter Studies as Topic/ (16891)

3 ((clinical or controlled or multicenter or multicentre or multi-center or multi-centre) adj2 (trial? or study or studies or research)).tw. (767858)

4 or/1-3 (955516)

5 Patient Selection/ (59037)

6 Patient Participation/ (22511)

7 Personnel Selection/ (12194)

8 (participat* or recruit* or enrol*).tw. (968575)

9 or/5-8 (1043466)

10 exp Informed Consent/ (38811)

11 (informed adj (consent or decision* or choice*)).tw. (38569)

12 or/10-11 (67122)

13 9 or 12 (1096693)

14 exp General Practice/ (71870)

15 General Practitioners/ (6148)

16 Physicians, Family/ (15790)

17 (family adj (practitioner? or practice? or doctor? or physician?)).tw. (24864)

18 (general adj (practitioner? or practice?)).tw. (72546)

19 or/14-18 (136820)

20 4 and 13 and 19 (2565)

**Source: Embase 1947-Present, updated daily**

Interface: OvidSP

Database coverage dates: 1947 to present

Search date: 23 May 2018

Retrieved records: 3545

Search strategy:

1 exp "clinical trial (topic)"/ (268156)

2 ((clinical or controlled or multicenter or multicentre or multi-center or multi-centre) adj2 (trial? or study or studies or research)).tw. (1110689)

3 or/1-2 (1236479)

4 patient selection/ (84060)

5 patient participation/ (23530)

6 personnel management/ (55756)

7 (participat* or recruit* or enrol*).tw. (1461388)

8 or/4-7 (1601441)

9 informed consent/ (93436)

10 (informed adj (consent or decision* or choice*)).tw. (74374)

11 or/9-10 (120989)

12 8 or 11 (1692683)

13 general practice/ (80065)

14 general practitioner/ (89915)

15 (family adj (practitioner? or practice? or doctor? or physician?)).tw. (31843)

16 (general adj (practitioner? or practice?)).tw. (96029)

17 or/13-16 (199931)

18 3 and 12 and 17 (3545)

**Source: Cochrane Central Register of Controlled Trials (CENTRAL): Issue 4 of 12, April 2018 & Cochrane Database of Systematic Reviews (CDSR): Issue 5 of 12, May 2018**

Interface: Cochrane Library/Wiley Interscience

Database coverage dates: Not available

Search date: 23 May 2018

Retrieved records: CENTRAL subset = 553 & CDSR subset = 15

Search strategy:

#1 MeSH descriptor: [Clinical Trials as Topic] explode all trees 57992

#2 MeSH descriptor: [Multicenter Studies as Topic] this term only 2039

#3 ((clinical or controlled or multicenter or multicentre or multi-center or multi-centre) near/2 (trial? or study or studies or research)):ti,ab 164485

#4 #1 or #2 or #3 209112

#5 MeSH descriptor: [Patient Selection] this term only 3646

#6 MeSH descriptor: [Patient Participation] this term only 1228

#7 MeSH descriptor: [Personnel Selection] this term only 54

#8 (participat* or recruit* or enrol*):ti,ab 175407

#9 #5 or #6 or #7 or #8 178120

#10 MeSH descriptor: [Informed Consent] explode all trees 672

#11 (informed next (consent or decision* or choice*)):ti,ab 8638

#12 #10 or #11 8965

#13 #9 or #12 182907

#14 MeSH descriptor: [General Practice] explode all trees 2636

#15 MeSH descriptor: [General Practitioners] this term only 220

#16 MeSH descriptor: [Physicians, Family] this term only 488

#17 (family next (practitioner? or practice? or doctor? or physician?)):ti,ab 839

#18 (general next (practitioner? or practice?)):ti,ab 4072

#19 #14 or #15 or #16 or #17 or #18 6628

#20 #4 and #13 and #19 595

**Source: OpenGrey**

Interface: http://www.opengrey.eu/

Database coverage dates: Not available

Search date: 1 June 2018

Retrieved records: 230 (filtered to English language)

Search strategy:

((family NEAR/1 (practitioner* or practice* or doctor* or physician*) OR (general NEAR/1 (practitioner* or practice*)))

**Source: National Technical Reports Library (NTIS)**

Interface: https://ntrl.ntis.gov/NTRL/

Database coverage dates: Not available

Search date: 1 June 2018

Retrieved records: 65

Search strategy:

(("family practitioner" or "family doctor" or "family physician" or "family practice" or "general practitioner" or "general practice") and (rct or "clinical trial" or "controlled trial" or "multicenter study" or "multi-center study") and (participat* or recruit* or enrol* or "informed consent" or "informed decision" or "informed choice"))

**Updated Search - 8 September 2020**

**Source: Ovid MEDLINE(R) In-Process & Other Non-Indexed Citations and Ovid MEDLINE**

Interface: OvidSP

Database coverage dates: 1946 to September 04, 2020

Search date: 8 September 2020

Retrieved records: 506

Search strategy:

1 exp Clinical Trials as Topic/ (345026)

2 Multicenter Studies as Topic/ (18579)

3 ((clinical or controlled or multicenter or multicentre or multi-center or multi-centre) adj2 (trial? or study or studies or research)).tw. (917282)

4 or/1-3 (1116345)

5 Patient Selection/ (65297)

6 Patient Participation/ (25972)

7 Personnel Selection/ (12877)

8 (participat* or recruit* or enrol*).tw. (1173504)

9 or/5-8 (1255851)

10 exp Informed Consent/ (41136)

11 (informed adj (consent or decision* or choice*)).tw. (45486)

12 or/10-11 (75383)

13 9 or 12 (1314867)

14 exp General Practice/ (75028)

15 General Practitioners/ (7936)

16 Physicians, Family/ (16393)

17 (family adj (practitioner? or practice? or doctor? or physician?)).tw. (26631)

18 (general adj (practitioner? or practice?)).tw. (79399)

19 or/14-18 (147067)

20 4 and 13 and 19 (2980)

21 ("20180523" or "20180524" or "20180525" or "20180526" or "20180527" or "20180528" or "20180529" or 2018053* or 201806* or 201807* or 201808* or 201809* or 20181* or 2019* or 202*).dt,ez,ed. (3617640)

22 20 and 21 (506)

**Source: Embase 1947-Present, updated daily**

Interface: OvidSP

Database coverage dates: 1947 to present

Search date: 8 September 2020

Retrieved records: 724

Search strategy:

1 exp "clinical trial (topic)"/ (331254)

2 ((clinical or controlled or multicenter or multicentre or multi-center or multi-centre) adj2 (trial? or study or studies or research)).tw. (1348844)

3 or/1-2 (1501706)

4 patient selection/ (94424)

5 patient participation/ (27583)

6 personnel management/ (58282)

7 (participat* or recruit* or enrol*).tw. (1814964)

8 or/4-7 (1968118)

9 informed consent/ (108837)

10 (informed adj (consent or decision* or choice*)).tw. (91543)

11 or/9-10 (142662)

12 8 or 11 (2074138)

13 general practice/ (85358)

14 general practitioner/ (103996)

15 (family adj (practitioner? or practice? or doctor? or physician?)).tw. (34703)

16 (general adj (practitioner? or practice?)).tw. (106481)

17 or/13-16 (221807)

18 3 and 12 and 17 (4202)

19 limit 18 to dc=20180523-20200908 (724)

**Source: Cochrane Central Register of Controlled Trials (CENTRAL): Issue 9 of 12, September 2020 & Cochrane Database of Systematic Reviews (CDSR): Issue 9 of 12, September 2020**

Interface: Cochrane Library/Wiley Interscience

Database coverage dates: Not available

Search date: 8 September 2020

Retrieved records: CENTRAL subset = 532 & CDSR subset = 16

Search strategy:

#1 MeSH descriptor: [Clinical Trials as Topic] explode all trees 47837

#2 MeSH descriptor: [Multicenter Studies as Topic] this term only 1881

#3 ((clinical or controlled or multicenter or multicentre or multi-center or multi-centre) near/2 (trial? or study or studies or research)):ti,ab 521338

#4 #1 or #2 or #3 549204

#5 MeSH descriptor: [Patient Selection] explode all trees 3466

#6 MeSH descriptor: [Patient Participation] this term only 1344

#7 MeSH descriptor: [Personnel Selection] this term only 50

#8 (participat* or recruit* or enrol*):ti,ab 292891

#9 #5 or #6 or #7 or #8 295260

#10 MeSH descriptor: [Informed Consent] explode all trees 712

#11 (informed next (consent or decision* or choice*)):ti,ab 56767

#12 #10 or #11 57085

#13 #9 or #12 329383

#14 MeSH descriptor: [General Practice] explode all trees 2434

#15 MeSH descriptor: [General Practitioners] this term only 269

#16 MeSH descriptor: [Physicians, Family] this term only 450

#17 (family next (practitioner? or practice? or doctor? or physician?)):ti,ab 1895

#18 (general next (practitioner? or practice?)):ti,ab 9791

#19 #14 or #15 or #16 or #17 or #18 12233

#20 #4 and #13 and #19 3130

#21 #4 and #13 and #19 with Publication Year from 2018 to 2020, with Cochrane Library publication date Between May 2018 and Sep 2020, in Trials 532

**Source: OpenGrey**

Interface: http://www.opengrey.eu/

Database coverage dates: Not available

Search date: 8 September 2020

Retrieved records: 230 (filtered to English language) [0 new]

Search strategy:

((family NEAR/1 (practitioner* or practice* or doctor* or physician*) OR (general NEAR/1 (practitioner* or practice*)))

**Source: National Technical Reports Library (NTIS)**

Interface: https://ntrl.ntis.gov/NTRL/

Database coverage dates: Not available

Search date: 8 September 2020

Retrieved records: 66 [1 new]

Search strategy:

(("family practitioner" or "family doctor" or "family physician" or "family practice" or "general practitioner" or "general practice") and (rct or "clinical trial" or "controlled trial" or "multicenter study" or "multi-center study") and (participat* or recruit* or enrol* or "informed consent" or "informed decision" or "informed choice"))
