# Supplementary material for: Factors associated with recruitment to randomised controlled trials in general practice: a systematic mixed studies review
Source: Trials. 2023 Feb 6;24:90. doi: 10.1186/s13063-022-06865-x (PMC9903494; doi:10.1186/s13063-022-06865-x)
Supplement: Supplementary file 3 — Additional file 3. Summary of study characteristics. [file 13063_2022_6865_MOESM3_ESM.docx]

| **Quantitative studies** | | | | | | | | | | | | |
| --- | --- | --- | --- | --- | --- | --- | --- | --- | --- | --- | --- | --- |
| **Article** | **Study design** | **Setting of parent RCT** | | | | | **Population** | | | **Intervention or variable assessed** | **Comparator** | **Outcome** |
|  |  | Lead country | Discipline | Sub-discipline | Intervention | Design (cluster or individual randomisation) | Recruitment focus (practice, practitioners, patient) | Study size | Participants/decliners/both |  |  |  |
| Markun et al, 2016 (1) | NRSI | Switzerland | Respiratory | COPD | Multifaceted training for PCPs and practice assistants | Cluster | Patients | 216 (71 by case-finding) | Participants | Opportunistic case-finding of patients with undiagnosed COPD | Patients recruited with known COPD | Total number of recruited |
| Powell et al, 2016 (2) | Cross-sectional survey | UK (England) | Dermatology | Eczema | four intervention groups by Emollient type | Individual | Patients | 197 | Participants | Self-referral recruitment | In-consultation recruitment | Total number recruited |
| Horspool et al, 2015 (3) | Cross-sectional survey | UK (England and Wales) | Respiratory  Paediatrics | Asthma | Letter from GP to remind parents/carers to maintain and renew medication | Cluster | Practice | 433 practices invited, 134 expressions of interest, 129 practices recruited. | Both | Practice size and previous research experience | N/A | Total number randomised |
| Jennings et al, 2015(4) | RCT | UK (Scotland), Denmark, Netherlands | Cardiovascular | Adverse drug-related CV effects | Switching to celecoxib | Individual | Patients (Scotland only) | 50 | Participants | £100 financial incentive | No incentive | Total number recruited and randomised |
| Warren et al, 2014 (5) | Feasibility RCT | UK (England) | Public Health | Exercise | two interventions relating to patient education and a pedometer | Individual and Cluster | Practice and Patient | 24 practices; 131 patients | Participants | Recruitment Strategy: opportunistic  Randomisation Strategy: individual | Recruitment Strategy: Systematic  Randomisation  Strategy:  Practice cluster | 1. Time to practice recruitment  2. Time to participant recruitment |
| Williams et al, 2014 (6) | Cross-sectional study | Australia | Musculoskeletal | Low back pain | two interventions arms using Paracetamol | Individual | Patients | 1,195 | Participants | Characteristics of GP and practice  Operational aspects of the study | N/A | Rate of recruitment (number recruited by number of days GP was recruiting for) |
| McLean et al, 2014 (7) | Cross-sectional study | New Zealand | Care of older adults | Unmet need/risk of decline | Brief Risk Identification Geriatric Health Tool (BRIGHT) | Cluster | Patients | 3893 | Participants | Characteristics of GP and Practices | N/A | Rate of recruitment (Recruited over those eligible) |
| Colwell et al, 2012 (8) | NRSI | UK (England) | Diabetology | Type 2 Diabetes | Patient Decision Aid | Cluster | Practices | 54 | Participants | Viral Marketing Techniques | Traditional recruitment | Total number of practices recruited |
| Fletcher et al, 2010 (9) | Time-series analysis | UK (England) | Cardiovascular | Atrial Fibrillation | Warfarin vs aspirin | Individual | Patients | 973 | Participants | Changes to design/conduct of trial | N/A | Recruitment rate per 1000 population |
| Ellis et al, 2007 (10) | NRSI | USA (North Carolina) | Cardiovascular | Cardiovascular risk | Personal digital assistant vs automated BP monitor | Cluster | Practices and practitioners | 68 practices, 184 (84%) practitioners responded to the survey | Participants | 10 different recruitment strategies | N/A | Recruitment as a total number and as a proportion of those contacted |
| Brealey et al, 2007 (11) | NRSI | UK | Musculoskeletal | Knee pathology | Direct access to MRI | Individual | Patients and practices | 553 patients 647 practices invited; 285 (44%) practices recruited | Participants (patients)  Participants and non-participants (practices) | Telephone randomisation of patients  Characteristics of practices | Postal randomisation | Total number of participants recruited |
| Fletcher et al, 2007 (12) | Cross-sectional study | UK (England and Wales) | Cardiovascular | Atrial Fibrillation | Warfarin vs aspirin | Individual | Patients | 1740 attended study appointment and eligible, 973 consented | Both | practice/practitioner and patient characteristics | N/A | Consent to participate in the study |
| Pearl et al, 2003 (13) | Cross-sectional survey | New Zealand | Cardiovascular | Heart Failure | Brain natriuretic peptide result | Individual | Patients and practitioner | 186 practitioners recruited of whom 84 recruited patients | Participants (those practitioners who did/did not refer patients) | 7 questions in a survey | N/A | N/A |
| Richardson et al, 2002 (14) | Cross-sectional survey | New Zealand | Gastrointestinal | Dyspepsia | *H.pylori* breath testing | Individual | Patients | 100 GPs (response rate 95 (97%)) | Participants | 6 Practice and practitioner characteristics | N/A | 1 or more patients enrolled vs no patients enrolled |
| Welsh et al, 2002 (15) | Cross-sectional study | USA (Minneapolis) | Obstetrics | Maternal infection/preterm labor | Vaginal pH testing | Individual | Patients | 310 patients eligible (71 recruited, 239 not recruited) 309 charts reviewed (1 chart missing from recruited) | Both | 5 participant characteristics | N/A | Enrolled/did not enroll |
| de Wit et al, 2001 (16) | Cross-sectional survey | Netherlands | Gastrointestinal | Dyspepsia | Cisapride vs Ranitidine | Individual | Patients and practitioners | 165 GP practitioners, 128 completed survey, 793 patients recruited. | Participants | 4 practice and practitioner characteristics | N/A | Total number of patients recruited |
| Durham et al, 1991 (17) | Cross-sectional study | USA | Public Health | Health Promotion | Not adequately specified | No protocol | Patients | 5,011 invited  2713 participants,  2208 non-participants | Both | 7 participant characteristics | N/A | Number of patients recruited and %age of those invited |

| **Qualitative Studies** | | | | | | | | | | |
| --- | --- | --- | --- | --- | --- | --- | --- | --- | --- | --- |
| **Article** | **Study Design** | **Setting of parent RCT** | | | | | **Population** | | | **Interest** |
|  |  | Lead country | Discipline | Sub-discipline | Intervention | Design (cluster or individual randomisation) | Recruitment focus | Study Size | Participants/ decliners/both |  |
| Flokstra-de Blok et al, 2018 (18) | Semi-structured interviews | Netherlands | Allergy | IgE-mediated allergy | Allergy Management Support system (AMSS) | Cluster | Patients | 10 GPs | Participants (GPs who did not recruit) | Why GPs who were part of the study did not recruit. |
| Van der Gaag, 2017 (18) | Open-ended survey question | Netherlands | MSK | Sciatica | Stepped up medication or immediate morphine | Individual | Patients | 22 GPs | Decliners | Determinants in the recruitment process leading to discontinuation of the trial. |
| Attwood et al, 2016 (19) | Semi-structured interview | UK (England) | Public Health | Physical Activity | Very brief pedometer intervention | Individual | Patients | 25 | Decliners | Reasons for not taking part in the trial. |
| Bleidorn et al, 2015 (20) | Semi-structured interview | Germany | Infectious disease | Urinary Tract Infection | Ibuprofen and conditional use of antibiotics | Individual | Patients | 20 | Participants | Motivation and barriers to participation |
| van Staa et al, 2014 (21) | In-depth interview | UK – England and Scotland | Cardiovascular and Respiratory (2 trials) | Cardiovascular risk and COPD respectively | Statin and antibiotics respectively | Individual | Practitioners and patients | 27 GP interviews (15 participants, 12 decliners) 10 patients | Participants (patients) and both (GPs) | Barriers and facilitators for GPs and patients |
| Maeland et al, 2011 (22) | Survey | Norway | Occupational health | Sick-leave certification | Sick leave vs not | Individual | Patients | 50 GPs | Neither | Reluctance to recruit patients for a sick leave RCT. |
| Dormandy et al, 2008 (23) | Informal interviews | UK - England | Obstetrics/Haematology | Antenatal sickle cell/thalassaemia screening | Screening in primary care | Cluster | Patients | 20 GPs | Participants | GP motivations for taking part in the trial |
| Salmon et al, 2007 (24) | Interview | UK - England | Medically unexplained symptoms | N/A | Peer learning and video feedback | Cluster | Practitioners | 16 GPs | Decliners | Barriers of taking part in the study |
| Prout et al, 2003 (25) | Semi-structured interview | UK - Wales | Infectious Disease/Respiratory | Upper Respiratory Tract infection | intranasal disodium cromoglycate | Individual | Practitioners | 9 GPs, 1 practice nurse | Participants | Accounts of taking part in a trial using opportunistic recruitment |
| Fairhurst et al, 1996 (26) | Semi-structured interview | UK – England | Mental Health | Counselling | Counselling | Individual | Patients | 8 GPs | Participants | Reasons for difficulties in recruiting patients |

| **Mixed-methods studies** | | | | | | | | | | | |
| --- | --- | --- | --- | --- | --- | --- | --- | --- | --- | --- | --- |
| **Article** | **Study Design** | **Setting of parent RCT** | | | | | **Population** | | | **Intervention, variable assessed, or interest** | **Outcome** |
|  |  | **Lead country** | **Discipline** | **Sub-discipline** | **Intervention** | **Design (cluster or individual randomisation)** | **Recruitment focus** | **Study size** | **Participants//decliners/**  **both** |  |  |
| Loskutova et al, 2018 (27) | Sequential explanatory | USA | Renal/Cardiovascular | Chronic Kidney Disease | Clinical Decision support + facilitation | Cluster | Practices | 114 – 25 participants, 89 non-participants (quant & qual) | Both (Quant), decliners (Qual) | Barriers in recruiting practices | Number of practices enrolled |
| Brodaty et al, 2013 (28) | Convergent | Australia | Mental Health | Dementia | Educational | Cluster | Practitioners | 30 – 20 participants, 10 refusers | Both | reasons for,  and barriers against, participation | N/A |
| Blair et al, 2017 (29) | Sequential Explanatory | UK – England | Infectious disease | Antimicrobial resistance | Multi-modal intervention including clinical decision support | Cluster | Patients | 28 clinicians (qual – semi-structured interview) | Participants | Recruitment inc differential recruitment | Numbers and proportions of patients recruited |
| Normansell et al, 2016 (30) | Sequential explanatory | UK (England) | Public Health | Physical Activity | 2 intervention arms including pedometer +/- support | Cluster (by household not practice) | Patients | 1140 survey, 30 interviewed | Decliners | Reasons for non-participation. | N/A |
| Foster et al, 2015 (31) | Survey | Australia | Respiratory | Asthma | 4 intervention groups relating to inhaler adherence | Professional-cluster | Patients | 55 GPs participated | Participants | Barriers and facilitators to patient recruitment | N/A |
| Rogers et al, 2014 (32) | Sequential explanatory | UK – England | Public Health | Physical Activity | Nurse-led exercise intervention | Cluster | Patients | 988 cross-sectional study (690 non-participants, 298 participants),  15 interviews | Both (cross-sectional study), decliners (qual – semi-structured interview) | Factors influencing recruitment (quant)  Reason for not participating (qual) | Number recruited |
| Page et al, 2011 (33) | Survey | Australia | MSK | Low back pain | Behaviour change intervention – guideline adherence | Cluster (practitioner) | Patients | 79 GPs (quant), 44 GPs (qual) | Both | Factors contributing to GPs’ recruitment of patients | N/A |
| Gunn et al, 2008 (34) | Survey | Australia | Public Health | Childhood overweight/obesity | 4 GP consultations re: weight loss | Individual | Practitioners | 30 | Participants | Why GPs took part in the trial | N/A |
| Shelton et al, 2002 (35) | cross-sectional study and interviews | USA | Cancer | Screening/counselling | Not stated and protocol not available | Cluster | Practitioners | 136 participants  59 decliners | Both | Practice and physician characteristics | Number of participants |
| Petty et al, 2001 (36) | Cross-sectional study and interviews | UK - England | Care of older adults | Prescribing | Pharmacist-run medication review | Individual | Patients | 2403 – 1214 consented, 1189 non-consented (quant), 68 (qual) | Both (quant), decliners (qual) | A number of patient factors. Quant  Reasons why patients declined to consent to the study | Number of patients recruited and rates |

Table 1: Summary of study characteristics

**References**

1. Markun S, Rosemann T, Dalla-Lana K, Steurer-Stey C. The impact of case finding on the recruitment yield for COPD research in primary care: An observational study. Respiration. 2016;92(5):308-15.

2. Powell K, Wilson VJ, Redmond NM, Gaunt DM, Ridd MJ. Exceeding the recruitment target in a primary care paediatric trial: an evaluation of the Choice of Moisturiser for Eczema Treatment (COMET) feasibility randomised controlled trial. Trials [Electronic Resource]. 2016;17(1):550.

3. Horspool MJ, Julious SA, Mooney C, May R, Sully B, Smithson WH. Preventing and Lessening Exacerbations of Asthma in School-aged children Associated with a New Term (PLEASANT): Recruiting Primary Care Research Sites-the PLEASANT experience. NPJ Primary Care Respiratory Medicine. 2015;25:15066.

4. Claudine G. Jennings TMM, Li Wei MJB, Lewis McConnachie IS. Does offering an incentive payment improve recruitment to clinical trials and increase the proportion of socially deprived and elderly participants? 2015;16(1):80.

5. Warren FC, Stych K, Thorogood M, Sharp DJ, Murphy M, Turner KM, Holt TA, Searle A, Bryant S, Huxley C, Taylor RS, Campbell JL, Hillsdon M. Evaluation of different recruitment and randomisation methods in a trial of general practitioner-led interventions to increase physical activity: a randomised controlled feasibility study with factorial design. Trials [Electronic Resource]. 2014;15:134.

6. Williams CM, Maher CG, Hancock MJ, McAuley JH, Lin CWC, Latimer J. Recruitment rate for a clinical trial was associated with particular operational procedures and clinician characteristics. Journal of Clinical Epidemiology. 2014;67(2):169-75.

7. McLean C, Kerse N, Moyes SA, Ng T, Lin SYS, Peri K. Recruiting older people for research through general practice: the Brief Risk Identification Geriatric Health Tool trial. Australas J Ageing. 2014;33(4):257-63.

8. Colwell B, Mathers N, Ng CJ, Bradley A. Improving recruitment to primary care trials: some lessons from the use of modern marketing techniques. British Journal of General Practice. 2012;62(602):496-8.

9. Fletcher K, Mant J, Roalfe A, Hobbs FDR. Impact of study design on recruitment of patients to a primary care trial: an observational time series analysis of the Birmingham Atrial Fibrillation Treatment of the Aged (BAFTA) Study. Family practice. 2010;27(6):691-7.

10. Ellis SD, Bertoni AG, Bonds DE, Clinch CR, Balasubramanyam A, Blackwell C, Chen H, Lischke M, Goff DC. Value of recruitment strategies used in a primary care practice-based trial. Contemporary Clinical Trials. 2007;28(3):258-67.

11. Brealey SD, Atwell C, Bryan S, Coulton S, Cox H, Cross B, Fylan F, Garratt A, Gilbert FJ, Gillan MG, Hendry M, Hood K, Houston H, King D, Morton V, Orchard J, Robling M, Russell IT, Torgerson D, Wadsworth V, Wilkinson C. Using postal randomization to replace telephone randomization had no significant effect on recruitment of patients. Journal of Clinical Epidemiology. 2007;60(10):1046-51.

12. Fletcher K, Mant J, Holder R, Fitzmaurice D, Lip GYH, Hobbs FDR. An analysis of factors that predict patient consent to take part in a randomized controlled trial. Family practice. 2007;24(4):388-94.

13. Pearl A, Wright S, Gamble G, Doughty R, Sharpe N. Randomised trials in general practice - A New Zealand experience in recruitment. New Zealand Medical Journal. 2003;116(1186).

14. Richardson A, Sutherland M, Wells E, Toop L, Plumridge L. Factors affecting general practitioner involvement in a randomised controlled trial in primary care. New Zealand Medical Journal. 2002;115(1151):153-5.

15. Welsh JL, Adam P, Fontaine P, Gjerdingen D. Recruiting for a randomized controlled trial from an ethnically diverse population: lessons from the Maternal Infection and Preterm Labor Study. J. 2002;51(9):760.

16. Wit NJd, Quartero AO, Zuithoff AP, Numans ME. Participation and successful patient recruitment in primary care. J. 2001;50(11):976.

17. Durham ML, Beresford S, Diehr P, Grembowski D, Hecht JA, Patrick DL. Participation of higher users in a randomized trial of Medicare reimbursement for preventive services. The Gerontologist. 1991;31(5):603-6.

18. Flokstra-de Blok BMJ, Brakel TM, Wubs M, Skidmore B, Kocks JWH, Oude Elberink JNG, Schuttelaar MA, van der Velde JL, van der Molen T, Dubois AEJ. The feasibility of an allergy management support system (AMSS) for IgE-mediated allergy in primary care. Clin Transl Allergy. 2018;8:18.

19. Attwood S, Morton KL, Mitchell J, Emmenis MV, Sutton S, Team VBIP. Reasons for non-participation in a primary care-based physical activity trial: a qualitative study. BMJ Open. 2016;6(5):e011577.

20. Bleidorn J, Bucak S, Gagyor I, Hummers-Pradier E, Dierks ML. Why do - or don't - patients with urinary tract infection participate in a clinical trial? A qualitative study in German family medicine. German Medical Science. 2015;13:Doc17.

21. Staa TPv, Dyson L, McCann G, Padmanabhan S, Belatri R, Goldacre B, Cassell J, Pirmohamed M, Torgerson D, Ronaldson S, Adamson J, Taweel A, Delaney B, Mahmood S, Baracaia S, Round T, Fox R, Hunter T, Gulliford M, Smeeth L. The opportunities and challenges of pragmatic point-of-care randomised trials using routinely collected electronic records: evaluations of two exemplar trials. Health technology assessment (Winchester, England). 2014;18(43):1-146.

22. Maeland S, Magnussen LH, Eriksen HR, Malterud K. Why are general practitioners reluctant to enrol patients into a RCT on sick leave? A qualitative study. Scand J Public Health. 2011;39(8):888-93.

23. Dormandy E, Kavalier F, Logan J, Harris H, Ishmael N, Marteau TM, Anionwu EN, Atkin K, Brown K, Bryan S, Calnan M, Davis V, Dick M, Gulliford M, Johnston T, Jones P, Karnon J, Reid EP, Roberts T, Wild B. Maximising recruitment and retention of general practices in clinical trials: A case study. British Journal of General Practice. 2008;58(556):759-66.

24. Salmon P, Peters S, Rogers A, Gask L, Clifford R, Iredale W, Dowrick C, Morriss R. Peering through the barriers in GPs' explanations for declining to participate in research: the role of professional autonomy and the economy of time. Family practice. 2007;24(3):269-75.

25. Prout H, Butler C, Kinnersley P, Robling M, Hood K, Tudor-Jones R. A qualitative evaluation of implementing a randomized controlled trial in general practice. Family practice. 2003;20(6):675-81.

26. Fairhurst K, Dowrick C. Problems with recruitment in a randomized controlled trial of counselling in general practice: causes and implications. J Health Serv Res Policy. 1996;1(2):77-80.

27. Loskutova NY, Smail C, Ajayi K, Pace WD, Fox CH. Recruiting primary care practices for practice-based research: a case study of a group-randomized study (TRANSLATE CKD) recruitment process. Family practice. 2018;35(1):111-6.

28. Brodaty H, Gibson LH, Waine ML, Shell AM, Lilian R, Pond CD. Research in general practice: a survey of incentives and disincentives for research participation. Ment Health Fam Med. 2013;10(3):163-73.

29. Blair PS, Turnbull S, Ingram J, Redmond N, Lucas PJ, Cabral C, Hollinghurst S, Dixon P, Peters T, Horwood J, Little P, Francis NA, Gilbertson A, Jameson C, Hay AD. Feasibility cluster randomised controlled trial of a within-consultation intervention to reduce antibiotic prescribing for children presenting to primary care with acute respiratory tract infection and cough. BMJ Open. 2017;7(5):e014506.

30. Normansell R, Holmes R, Victor C, Cook DG, Kerry S, Iliffe S, Ussher M, Fox-Rushby J, Whincup P, Harris T. Exploring non-participation in primary care physical activity interventions: PACE-UP trial interview findings. Trials [Electronic Resource]. 2016;17:178.

31. Foster JM, Sawyer SM, Smith L, Reddel HK, Usherwood T. Barriers and facilitators to patient recruitment to a cluster randomized controlled trial in primary care: lessons for future trials. BMC medical research methodology. 2015;15:18.

32. Rogers A, Harris T, Victor C, Woodcock A, Limb E, Kerry S, Iliffe S, Whincup P, Ekelund U, Beighton C, Ussher M, Adams F, Cook DG. Which older people decline participation in a primary care trial of physical activity and why: insights from a mixed methods approach. BMC geriatrics. 2014;14:9.

33. Page MJ, French SD, McKenzie JE, Connor DAO, Green SE. Recruitment difficulties in a primary care cluster randomised trial: investigating factors contributing to general practitioners' recruitment of patients. BMC medical research methodology. 2011;11:35.

34. Gunn J, McCallum Z, Sanci L. What do GPs get out of participating in research? - experience of the LEAP trial. Australian family physician. 2008;37(5):372-5.

35. Shelton BJ, Wofford JL, Gosselink CA, McClatchey MW, Brekke K, Conry C, Wolfe P, Cohen SJ. Recruitment and retention of physicians for primary care research. J Community Health. 2002;27(2):79-89.

36. Petty DR, Zermansky AG, Raynor DK, Vail A, Lowe CJ, Freemantle N, Buttress AD. "No thank you": why elderly patients declined to participate in a research study. Pharm World Sci. 2001;23(1):22-7.
