# Supplementary material for: Factors associated with recruitment to randomised controlled trials in general practice: a systematic mixed studies review
Source: Trials. 2023 Feb 6;24:90. doi: 10.1186/s13063-022-06865-x (PMC9903494; doi:10.1186/s13063-022-06865-x)
Supplement: Supplementary file 4 — Additional file 4. Assessment of methodological limitations. [file 13063_2022_6865_MOESM4_ESM.docx]

| Study ID | Aims | Design | Size | Pop | Sample | Selection | Non-response | Outcome | Measurement | Statistical Significance | Reproducibility | Data | Response rate* | Non-esponders | Consistent | Results | Conclusion | Limitations | Conflicts* | Ethics | Overall assessment |
| --- | --- | --- | --- | --- | --- | --- | --- | --- | --- | --- | --- | --- | --- | --- | --- | --- | --- | --- | --- | --- | --- |
| Powell 2016 | Y | Y | Y | Y | Y | U | N | Y | U | Y | Y | N | U | N | U | U | Y | Y | N | Y | Major concerns |
| Horspool 2015 | Y | Y | Y | Y | Y | Y | N/A | Y | Y | Y | Y | N | N/A | N/A | Y | Y | Y | U | N | Y | Minor concerns |
| Williams 2014 | Y | Y | Y | Y | Y | Y | U | Y | Y | Y | Y | Y | U | U | Y | N | Y | Y | U | Y | Minor concerns |
| McLean 2014 | Y | Y | Y | Y | Y | Y | N | Y | N | Y | N | Y | Y | N | Y | Y | Y | Y | U | Y | Major concerns |
| Fletcher 2007 | Y | Y | Y | Y | Y | Y | Y | Y | Y | Y | Y | Y | N | Y | Y | Y | Y | Y | N | Y | No concerns |
| Pearl 2003 | N | Y | Y | Y | Y | Y | N | Y | N | Y | Y | Y | Y | N | Y | Y | Y | Y | N | Y | Major concerns |
| Richardson 2002 | Y | Y | Y | Y | Y | Y | Y | Y | Y | Y | N | Y | N | N/A | Y | Y | Y | N | U | U | Major concerns |
| Welsh 2002 | Y | Y | Y | U | Y | Y | N/A | Y | Y | Y | N | U | N | N/A | U | Y | Y | N | U | Y | Major concerns |
| De Wit 2001 | Y | Y | Y | Y | Y | Y | N | Y | Y | Y | N | U | N | N | Y | Y | Y | N | U | Y | Major concerns |
| Durham 1991 | Y | Y | Y | Y | Y | Y | N/A | Y | Y | Y | Y | Y | N/A | N/A | N | N | Y | N | U | U | Major concerns |

**Quantitative: Cross-sectional**

*These items score poorly if answered yes.

**Quantitative: Non-randomised studies of interventions**

| Study ID | Confounding | Selection of Participants | Classification of interventions | Deviations from intended interventions | Missing data | Measurement of outcomes | Selection of the reported results | Overall Bias |
| --- | --- | --- | --- | --- | --- | --- | --- | --- |
| Markun 2016 | Serious | Low | Low | Low | Low | Low | Low | Serious |
| Fletcher 2010 | Critical | Low | Serious | NI | Low | Moderate | Low | Critical |
| Colwell 2012 | Critical | Low | Low | Low | Low | Serious | Low | Critical |
| Ellis 2007 | Critical | NI | Serious | Critical | Moderate | Serious | Low | Critical |
| Brealey 2007 | Serious | Low | Low | Low | Low | Moderate | Low | Serious |

**Quantitative: Randomised controlled trial**

| Study ID | Randomisation process | Timing of randomisation | Deviations from the intended interventions | Missing outcome data | Measurement of the outcome | Selection of the reported result | Overall Bias |
| --- | --- | --- | --- | --- | --- | --- | --- |
| Jennings 2015 | Low | N/A (individual randomisation) | Some concerns | Low | Some concerns | Low | Some concerns |
| Warren 2014 | Low | Low | Some concerns | Low | Low | Low | Some concerns |

**Qualitative**

| Study ID | Was there a clear aims statement? | Is a qualitative methodology appropriate? | Was the research design appropriate to address the aims of the research? | Was the recruitment strategy appropriate to the aims of the research? | Was the data collected in a way that addressed the research issue? | Has the relationship between researcher and participants been adequately considered? | Have ethical issues been taken into consideration? | Was the data analysis sufficiently rigorous? | Is there a clear statement of findings? | How valuable is the research? | Overall assessment |
| --- | --- | --- | --- | --- | --- | --- | --- | --- | --- | --- | --- |
| De Blok 2018 | Yes | Yes | Yes | Yes | Unclear | No | Yes | No | Yes | Yes | Major concerns |
| Van der Gaag 2017 | Yes | Yes | Yes | Unclear | Unclear | No | No | No | Yes | Yes | Major concerns |
| Blair 2017 | No | Yes | Yes | Yes | Yes | No | Yes | No | No | Yes | Major Concerns |
| Attwood 2016 | Yes | Yes | Yes | Yes | Yes | No | Yes | No | Yes | Yes | Major concerns |
| Bleidorn 2015 | Yes | Yes | Yes | Yes | Yes | Yes | Yes | Yes | Yes | Yes | No concerns |
| Van Staa 2014 | Yes | Yes | Yes | Yes | Yes | No | Yes | Yes | Yes | Yes | No concerns |
| Maeland 2011 | Yes | Yes | Yes | Yes | Yes | Yes | Yes | Yes | Yes | Yes | No Concerns |
| Dormandy 2008 | Yes | Yes | Yes | Yes | Unclear | No | No | No | No | Yes | Major concerns |
| Salmon 2007 | Yes | Yes | Yes | Yes | Yes | Unclear | Yes | Yes | Yes | Yes | No concerns |
| Prout 2003 | Yes | Yes | Yes | Unclear | Yes | Yes | No | Unclear | Yes | Yes | Minor concerns |
| Fairhurst 1996 | Yes | Yes | Yes | Unclear | Unclear | No | No | No | Yes | Yes | Major concerns |

**Mixed Methods**

| Study ID | Qualitative | | | | | | Quantitative | | | Mixed methods | | | Overall | |
| --- | --- | --- | --- | --- | --- | --- | --- | --- | --- | --- | --- | --- | --- | --- |
|  | Qualitative question? | Appropriate method? | Description of the context? | Description of participants and justification of sampling? | Description of qualitative data collection and analysis? | Discussion of researchers’ reflexivity | Appropriate sampling and sample? | Justification of measurements? | Control of confounding variables | Justification of the mixed methods design | Combination of qualitative and quantitative data collection-analysis | Combination of quant/qual data or results |  |  |
| Loskutova 2018 | Yes | Yes | Yes | No | No | Yes | No | Yes | No | Yes | Yes | Yes | Major concerns |  |
| Normansell 2016 | Yes | Yes | Yes | Yes | Yes | No | Yes | Yes | N/A | Yes | Yes | Yes | Minor concerns |  |
| Foster 2015 | Yes | Yes | Yes | No | Yes | Unclear | No | Yes | Yes | No | No | No | Major concerns |  |
| Rogers 2014 | Yes | Yes | Yes | Yes | Yes | Yes | Unclear | Yes | Yes | Yes | Yes | Yes | Minor concerns |  |
| Brodaty 2013 | Yes | No | Yes | Yes | No | No | No | Yes | No | Yes | Unclear | Yes | Major concerns |  |
| Page 2011 | Yes | Yes | Yes | Yes | Yes | Unclear | Yes | Yes | N/A | Yes | Yes | Yes | No concerns |  |
| Gunn 2008 | Yes | Yes | Yes | Yes | No | No | Yes | Yes | N/A | Yes | No | No | Major concerns |  |
| Shelton 2002 | Yes | Yes | Yes | No | No | No | No | Yes | No | No | No | No | Major concerns |  |
| Petty 2001 | Yes | Yes | Yes | No | No | No | Yes | Unclear | Unclear | No | No | No | Major concerns |  |

Table 2: Assessment of methodological limitations
