# Supplementary material for: Factors associated with recruitment to randomised controlled trials in general practice: a systematic mixed studies review
Source: Trials. 2023 Feb 6;24:90. doi: 10.1186/s13063-022-06865-x (PMC9903494; doi:10.1186/s13063-022-06865-x)
Supplement: Supplementary file 5 — Additional file 5. Quality appraisal summary. [file 13063_2022_6865_MOESM5_ESM.docx]

***Fig 1.*** *Summary of study quality. This figure illustrates the number of studies scored as having no, minor, moderate or major concerns. NB: This aggregates different study types.*

***Fig 2.*** *Cross-sectional study quality appraisal summary. This figure illustrates the aggregate scores of studies using the AXIS tool.*

***Fig 3.*** *RCT risk of bias summary. This figure illustrates the aggregate scores of RCTs using the RoB-2.0 tool. Abbreviation: RCT Randomised Controlled Trial; RoB Risk of Bias.*

***Fig 4.*** *NRSI risk of bias summary. This figure illustrates the aggregate scores of NRSIs using the* ROBINS-I tool*. Abbreviation: NRSI* Non-randomised Studies of Interventions*; ROBINS-I Risk Of Bias In Non-randomised Studies of Interventions.*

***Fig 5.*** *Qualitative quality appraisal summary. This figure illustrates the aggregate scores of qualitative studies using the CASP qualitative study checklist. Abbreviation: CASP Critical Appraisal Skills Programme.*

***Fig 12.*** *Mixed methods quality appraisal summary. This figure illustrates the aggregate scores of mixed methods studies using the mixed methods scoring system. Abbreviation: CASP Critical Appraisal Skills Programme.*
