# Supplementary material for: Factors associated with recruitment to randomised controlled trials in general practice: a systematic mixed studies review
Source: Trials. 2023 Feb 6;24:90. doi: 10.1186/s13063-022-06865-x (PMC9903494; doi:10.1186/s13063-022-06865-x)
Supplement: Supplementary file 6 — Additional file 6. Quantitative results tabulation. [file 13063_2022_6865_MOESM6_ESM.docx]

| **Factor** | **Paper** | **Variable measure** | **Outcome measure** | **Basic summary of variable** | **Summary of outcome** | **Statistical analysis** |
| --- | --- | --- | --- | --- | --- | --- |
| **Patient Factors** | | | | | | |
| Age | Fletcher 2007 | Age category | Number of patients giving consent n (%) | No raw data | Number (%)  75-79; 385 (59)  80-84; 395 (59);  85+; 193 (49) | OR (95% CI)  75-79; 1.00  80-84; 0.98 (0.79-1.22) 85+; 0.68 (0.53-0.88) |
|  | Durham 1991 | Mean age | Number of patients giving consent n (%) | No raw data | 65-69; 941 (59.4)  70-74; 856 (56.8)  75-79; 567 (53.9)  80-84; 247 (44.8)  85-69; 92 (39.2)  90 +; 12 (15.0) | ‘Participating enrollees were significantly younger’(table 1. unclear re: significance level for age) |
|  | Petty 2001 | Age category (number invited) | Number of patients giving consent n (%) | 65-74; 1305  75+; 1098 | 65-74; 736 (56)  75+; 453 (41) | OR (95% CI), p-value  1.0  0.54 (0.46-0.64), p < 0.0001 |
|  | Rogers 2014 | Age category (number invited) | Number of patients participating n (%) | 60-64; 367  65-69; 326  70-75; 295 | 60-64; 113 (37.9)  65-69; 104 (34.9)  70-75; 81 (27.2) | OR (95% CI), p-value  1.0  1.05 (0.76, 1.45)  0.85 (0.61, 1.19), p = 0.38 |
| Sex | Fletcher 2007 | Male/Female | Number of patients giving consent n (%) | No raw data | Male 530 (60)  Female 443 (58) | OR (95% CI)  Female 1.00  Male 1.09 (0.09-1.33) |
|  | Durham 1991 | Male/Female | Number of patients giving consent n (%) | No raw data | Male 1054 (55.7)  Female 1661 (53.3) | No significant difference. |
|  | Petty 2001 | Male/Female (number invited) | Number of patients giving consent n (%) | M 968  F 1435 | M 530 (55)  F 659 (46) | OR (95% CI), p-value  M 1.0  F 0.74 (0.63 - 0.88), p = 0.0005 |
|  | Rogers 2014 | Female/Male (number invited) | Number of patients participating n (%) | F 482  M 506 | F 160 (53.7)  M 138 (46.3) | OR (95% CI), p-value  F 1.0  M 0.75 (0.57, 0.99), p = 0.04 |
| Deprivation | Fletcher 2007 | Index of Multiple Deprivation | Number of patients giving consent n (%) | No raw data | 1 338 (54)  2 224 (54)  3 192 (52)  4 150 (65) | OR (95% CI)  1.00  0.99 (0.77-1.26)  0.97 (0.71-1.19)  1.55 (1.14-2.12) |
|  | Rogers 2014 | National quintiles of Index of Multiple Deprivation Rank, 1 = most deprived (number invited) | Number of patients participating n (%) | 1; 14  2; 41  3; 99  4; 191  5; 643 | 1; 2 (0.7)  2; 9 (3.0)  3; 18 (6.0)  4; 51 (17.1)  5; 218 (73.2) | OR (95%), p value  1; 0.32 (0.07, 1.46)  2; 0.55 (0.26, 1.17)  3; 0.43 (0.25,0.74)  4; 0.71 (0.50. 1.02)  5; 1.0  p <0.001 |
| Ethnicity | Fletcher 2007 | White/Non-white | Number of patients giving consent n (%) | No raw data | 913 (56)  29 (64) | OR (95% CI)  1.00  1.44 (0.77–2.67) |
|  | Welsh 2002 | Ethnicity (table unavailable showing ethnicities compared) | Enrolling vs declining to enrol | Data table unavailable | Data table unavailable | p = 0.042 |
| Migrant population | Welsh 2002 | Immigrants or non-immigrants | Enrolling vs declining to enrol. | Data table unavailable | Enrolling 12%  Declining enrolment 88% | p = 0.014 |
| Comorbidity | Durham 1991 | Chronic Disease Score | Mean score for consented and not consented patients | No raw data | Consented 2.7  Not consented 2.8 | T = 1.0; p = 0.05). |
|  | Fletcher 2007 | Rankin Disability Score (least to most disabled) | Number of patients giving consent n (%) | No raw data | 0; 173 (54)  1; 265 (58)  2; 296 (60)  3; 187 (53)  4-5; 26 (45) | OR (95% CI)  1.00  1.19 (0.89-1.58)  1.29 (0.97-1.71)  0.97 (0.72-1.31)  0.70 (0.40-1.22) |
| Polypharmacy | Fletcher 2007 | On 5 or more drugs | Number of patients giving consent n (%) | No raw data | No 559 (55)  Yes 414 (57) | OR (95% CI)  1.00  1.07 (0.89–1.30) |
|  | Petty 2001 | 1-4 or 5+ repeat medications (number invited) | Number of patients giving consent n (%) | 1-4; 1371  5+; 1032 | 1-4; 649 (47)  5+ 540 (52) | OR (95% CI), p-value  1-4; 1.0  5+; 1.3 (1.1 – 1.5), p = 0.002 |
| Proximity to clinic | Durham 1991 | Home zipcode same as clinic (Yes/No) | Number of patients giving consent n (%) | No raw data | Yes 1253 (57.2)  No 1462 (51.9) | Chi-squared significant at 0.001 level |
| Language barriers | Welsh 2002 | Translator requirement (number invited) | Number of patients enrolling n (%) | Translator required 71 | 1 (1.4%) | p = 0.008 |
| Disinterest of potential participants | Foster 2015 | ‘I approached patients…but they were not interested’ (number in each group) | Recruiter GPs compared with non-recruiter GPs (mean Likert scale score +/- SD) | Recruiter 37  Non-recruiter 5 | Recruiter; 4.3 ± 1.6  Non-recruiter 5.4 ± 1.3 | p = 0.193 |
|  | Page 2011 | ‘…I approached  patients to  participate…  but they were not interested (number of practice responses) | 7-point Likert-scale, 1 Strongly disagree; 7 Strongly agree (mean score) | 67 | 3.5 | 95% CI = 3.1-3.9 |
| Patient eligibility | Foster 2015 | ‘I screened patients…but they were not eligible’ (number in each group) | Recruiter GPs compared with non-recruiter GPs (mean Likert scale score +/- SD) | Recruiter 37  Non-recruiter 5 | Recruiter; 5.0 ± 1.3  Non-recruiter; 6.0 ± 1.0 | p = 0.128 |
|  | Foster 2015 | ‘I did not see any patients who would have been eligible’ (number in each group) | Recruiter GPs compared with non-recruiter GPs (mean Likert scale score +/- SD) | Recruiter 37  Non-recruiter 5 | Recruiter; 2.1 +/- 1.6  Non-recruiter 3.8 +/- 2.3 | p = 0.073 |
|  | Page 2011 | ‘I did not see any patients with acute non-specific low-back pain who would have been eligible for the study’ (number of practice responses) | 7-point Likert-scale, 1 Strongly disagree; 7 Strongly agree (mean score) | 66 | mean 2.7 | 95% CI = 2.3-3.1 |
| **Practice Factors** | | | | | | |
| Population size | Warren 2014 | List size (small <3500, medium 3500-8000, large >8000), number of practices in study | Mean Time to practice EOI | Small 7  Medium 8  Large 9 | Small 40.7  Medium 26.9  Large 29.6 | Between group mean difference (95% CI)  S vs M 13.8 (-8.1; 35.8)  S vs L 11.2 (-8.0; 30.4)  M vs L -2.7 (-18.6; 13.3) |
|  | Horspool 2015 | Practice size (1,000’s) | Number of Practice EOIs  Number of Practices randomised | No raw data | EOIs, n = 134  Randomised, n = 129 | Hazard ratio (95% CI); p-value  EOI: 1.03 (0.99-1.07); 0.122  Randomised: 1.04 (0.99–1.08); 0.137 |
| Age profile | McLean 2014 | Proportion of practice patients over 75 years (0-5; 5.1-10; 11-19) | Recruitment rate (%) of the practice | 0-5; 19 (39)  5.1-10; 16 (33)  11-19; 14 (29) | 0-5; 46.3  5.1-10; 46.9  11-19; 49.2 | Beta (P-value)  0.2659 (0.8169) |
| Deprivation | Warren 2014 | Practice deprivation score IMD 2007 (least; central two; most deprived quartile) | Mean Time to practice EOI, days | Least 3  Central 15  Most 6 | Least 25.0  Central 33.6  Most 31.2 | Between group mean difference (95% CI) L vs C -8.6 (-20.0; 2.8)  L vs M -6.2 (-24.2; 11.9)  C vs M 2.4 (-17.1; 22.0) |
|  | Foster 2015 | GPs practicing in location of social disadvantage (SEIFA quintile <3) | Recruiting and non-recruiting GPs, n (%) | N/A | Recruiting GPs 21 (53)  Non-recruiting GPs 9 (60) | Backward linear regression B-coefficient  -0.482; 95% CI -0.41 to -0.11 (model includes age and gender) |
|  | Williams 2014 | Socioeconomic status (SES) of suburb of GP clinic (high, moderate, low) | Rate of patients successfully recruited to the trial by each GP | SES percentile 75 +/- 26 (SD) | Not reported | Multivariate analysis, IRR (95% CI)  Comparison with low SES  Moderate SES 0.71 (0.50, 1.01)  High SES 0.52 (0.37, 0.74) |
| Rurality | Brealey 2007 | Practice distance from hospital | Number of patients recruited | Not reported | Not reported | Negative binomial regression  Coefficient; p-value;  -0.019; p = 0.001  e^b 0.981 (95% CI = 0.969 to 0.992) |
| Size of practice | Fletcher 2007 | Number of GPs (1-2; 3-4; 5-6; 7-8; >8) | Number of patients giving consent n (%) | No raw data | 1-2; 98 (75)  3-4; 262 (62)  5-6; 335 (54)  7-8; 225 (51)  >8; 53 (42) | Univariable logistic regression: coefficient (95% CI)  1.00  0.55 (0.36–0.86)  0.40 (0.26–0.61)  0.34 (0.22–0.53)  0.25 (0.15–0.42) |
|  | McLean 2014 | Number of GPs (Solo; 2-4; >5), n (%) | Recruitment rate of the practice (%) | Solo; 6 (10)  2-4; 30 (51)  >5; 23 (39) | 1; 46.4  2-5; 46.3  5-15; 48.2 | Beta (p=value)  0.0105 (0.54) |
|  | Brealey 2007 | Number of GPs working in the practice | Number of patients recruited | Median number of GPs 4 in participating and non-participating practices | Not reported | Negative binomial regression  Coefficient; p-value;  0.117; 0.001 |
|  | Richardson 2002 | Number of GPs working in the practice (1-2; 3-5; >5) | Number of practice respondents | 30 (32)  39 (41)  26 (27) | Not reported | States not associated but no data |
|  | Shelton 2002 | Number of GPs working in a solo or group practice (number of GPs invited) | Number of GP participants n (%) | Solo – 119  Group - 76 | Solo; 81 (60)  Group; 55 (40) | p = 0.524 |
|  | Loskutova 2018 | Health Care Organisation size, small or large. Number invited | Enrolled organisation n (%) | Small 33  Large 15 | Small 16 (48%)  Large 3 (20%) | OR 3.67, p = 0.11 |
| Research experience | Horspool 2015 | Number of previous RCTs | Number of Practice EOIs  Number of Practices randomised | No raw data | Not reported | HR (95%CI)  1.98 (1.57–2.51)  1.91 (1.52–2.42) |
|  | Horspool 2015 | Number of previous individual RCTs | Number of Practice EOIs  Number of Practices randomised | No raw data | Not reported | HR (95%CI)  2.36 (1.86–2.99)  2.36 (1.85–3.02) |
|  | Horspool 2015 | Number of previous cluster RCTs | Number of Practice EOIs  Number of Practices randomised | No raw data | Not reported | HR (95%CI)  1.91 (1.52–2.40)  1.83 (1.45–2.32) |
|  | Powell 2016 | Practice NIHR CRN Research level 1, 2 or sessional | Number recruited by in consultation referral | Level 1 – 8  Level 2 – 8  Sessional - 8 | Not reported | P = 0.116 |
| **Practitioner Factors** |  | | | | | |
| Age | Richardson 2002 | Age (30-39; 40-49; 50-59; 60-69) | Number of practice respondents | 22 (23)  49 (52)  21 (22)  3 (3) | Not reported | ‘not associated’ |
| Sex | Williams 2014 | Female n (%) | Rate of patients successfully recruited to the trial by each GP | 142 (39.5) | Not reported | Univariate analysis, coefficient (95% CI)  -0.44 (-0.88, -0.0001)  Not independently associated with recruitment |
|  | McLean 2014 | Recruitment rate by sex, n (%) | Recruitment rate (%) of the practice | Male 55 (43)  Female 73 (57) | Male 47.4  Female 46.9 | Beta(p-value)  -0.0051 (0.9422) |
|  | Richardson 2002 | Sex, n (%) | Number of practice respondents | Male 50 (53%)  Female 45 (47%) | Not reported | ‘not associated’ |
|  | Shelton 2002 | Male/Female  (number of GPs invited) | Number of GP participants n (%) | Male 174  Female 21 | Male 118 (87)  Female 18 (13) | p = 0.130 |
| Rurality | Shelton 2002 | Rural/Urban  (number of GPs invited) | Number of GP participants n (%) | Rural 81  Urban 114 | Rural 63 (46)  Urban 73 (54) | p = 0.040 |
| Clinical experience | McLean 2014 | Years practicing as a GP (in years) | Recruitment rate (%) of the practitioner | 0-10; 31 (28)  11-20; 43 (38)  21-40; 38 (34) | <10; 42.9  >10 47.5 | Beta (p-value)  0.0102 (0.0250) |
|  | Fletcher 2007 | Year of full GMC registration (<1975; 1976-1980; 1981-1985; 1986-1990; >1991) | Number (%) giving consent | Not reported | 195 (63)  206 (53)  195 (54)  238 (62)  139 (47) | OR (95%CI)  1.00  0.67 (0.49–0.91)  0.70 (0.51–0.95)  0.96 (0.71–1.31)  0.53 (0.39–0.74) |
|  | Williams 2014 | Average Years of Practice | Rate of patients successfully recruited to the trial by each GP | 21.7 | Not reported | Univariate analysis, coefficient (95% CI)  -0.024 (-0.04, -0.006)  Not independently associated |
| Duration of time at current practice | McLean 2014 | Length of time at that practice (1-5; 6-15; 16-40), n (%) | Recruitment rate (%) of the practitioner | 1-5; 34 (30)  6-15; 37 (33)  16-40; 41 (37) | Not reported | Did not include in GLM since no significant association. |
| Working Pattern | Richardson 2002 | Number of half days worked per week <5; 5-9; >9 | Number of practice respondents | 7 (7)  35 (37)  53 (56) | Not reported | “No association” |
| Professional Membership | Richardson 2002 | Member of Pegasus Independent (Y/N) Practitioner Association (IPA) | Number of practice respondents | 69 (73)  26 (27) | Not reported | “No association”  X2 = 1.35, p = 0.25 |
|  | Williams 2014 | RACGP fellow (n) | Rate of patients successfully recruited to the trial by each GP | 154 (42.4%) | Not reported | Univariate analysis, coefficient (95% CI)  -0.27 (-0.73, 0.19)  Not independently associated |
| Country of Training | McLean 2014 | Trained in New Zealand | Recruitment rate (%) of the practitioner | NZ; 84 (67) | 48.4% | Beta (p-value)  0.2257 (0.0210) |
| Engagement with Trial | Williams 2014 | GP readily contactable (contactable on more than half of attempts) | Rate of patients successfully recruited to the trial by each GP | 0.64 (0.24) | Not reported | Univariate analysis, coefficient (95% CI)  1.34 (0.90, 1.78) p<0.001  Multivariate analysis,  IRR (95% CI)  1.50 (1.07, 2.10) |
|  | Williams 2014 | GP routinely screens patients (returns >/1 ineligible screening form per month) | Rate of patients successfully recruited to the trial by each GP | 27% | Not reported | Univariate analysis, coefficient (95% CI), p- value  1.89 (1.50, 2.27), p <0.001  Multivariate analysis, IRR (95% CI)  2.08 (1.59, 2.94) |
|  | Durham 1991 | MD Planning (as proxy for engagement) | Number of patients giving consent n (%) | Not reported | Yes 506 (58.6) No 2,209 (53.3) | Chi squared significant at 0.05 level |
| Recruitment study engagement | Richardson 2002 | Responsiveness to recruitment survey invite (responded following 1^st^ invite or >/2 invites) | GPs enrolling ≥ 1 patient, number (%) | 1 mail-out; 55  ≥2 mail-outs; 40 | 1 mail-out; 31 (56)  ≥2 mail-outs; 11 (27.5) | Chi-squared 7.82; p = 0.005 |
| Forgetfullness | Page 2011 | ‘During the study period, I forgot to approach patients…’ (number of practice responses) | 7-point Likert-scale, 1 Strongly disagree; 7 Strongly agree (mean score) | 67 | 4.4 | 95% CI = 4.0 - 4.8 |
|  | Foster 2015 | ‘I forgot to approach patients…to participate’ (number in each group) | Recruiter GPs compared with non-recruiter GPs (mean Likert scale score +/- SD) | Recruiter 37  Non-recruiter 5 | Recruiter 2.7 ± 1.3  Non-recruiter 1.8 ± 0.4 | p = 0.133 |
| Intention to recruit | Foster 2015 | ‘I intended to approach patients’ (number in each group) | Recruiter GPs compared with non-recruiter GPs (mean Likert scale score +/- SD) | Recruiter 37  Non-recruiter 5 | Recruiter 6.1 ± 1.2  Non-recruiter 5.4 ± 0.9 | p = 0.128 |
|  | Page 2011 | ‘I intended to approach eligible patients…’ (number of practice responses) | 7-point Likert-scale, 1 Strongly disagree; 7 Strongly agree (mean score) | 67 | 5.6 | 95% CI = 5.2 - 5.9 |
| Readiness to change | Shelton 2002 | Readiness score, higher implies more ready to change (number of GPs) | GP participants/GP non-participants (mean readiness score) | Participants 136  Non-participants 59 | Participants 22.5  Non-participants 21.9 | p = 0.664 |
| Altruism | Brodaty 2013 | ‘Altruism/desire to contribute to research’ survey item as motivating factor for (possible) participation, total number responding | Number (%) of GPs answering in treatment, control, refuser groups and total | Treatment 10  Control 10  Refuser 10  Total 30 | Treatment 2 (20)  Control 8 (80)  Refuser 8 (80)  Total 18 (60) | Not conducted |
|  | Ellis 2007 | ‘Desire to help my colleagues’ survey item as reason for participating, total number responding | Number (%) of GPs answering | 184 | 27 (14) | Not conducted |
| Collaboration | Brodaty 2013 | ‘Collaborate with other professionals/form or strengthen contacts’ survey item as motivating factor for (possible) participation, total number responding | Number (%) of GPs answering in treatment, control, refuser groups and total | Treatment 10  Control 10  Refuser 10  Total 30 | Treatment 3 (30)  Control 2 (20)  Refuser 5 (50)  Total 10 (33) | Not conducted |
|  | Gunn 2008 | ‘I enjoyed collaborating with other professionals (both GPs and non-GPs)’ survey item as degree to which this influenced decision to participate, total number responding | Number (%) of GPs answering survey item by very, quite, a little, not at all agreement. | 29 | Very 12 (41)  Quite 10 (34)  A little 4 (14)  Not at all 3 (10) | Not conducted |
| CME points | Brodaty 2013 | ‘Fulfil CME requirements’ survey item as motivating factor for (possible) participation, total number responding | Number (%) of GPs answering in treatment, control, refuser groups and total | Treatment 10  Control 10  Refuser 10  Total 30 | Treatment 5 (50)  Control 4 (40)  Refuser 4 (40)  Total 13 (43) | Not conducted |
|  | Gunn 2008 | ‘it enabled me to fulfil my CME requirements’ survey item as degree to which this influenced decision to participate, total number responding | Number (%) of GPs answering survey item by very, quite, a little, not at all agreement. | 29 | Very 4 (14)  Quite 8 (28)  A little 14 (48)  Not at all 3 (10) | Not conducted |
|  | Ellis 2007 | ‘Ability to earn convenient CME credit’ survey item as reason for participating, total number responding | Number (%) of GPs answering | 184 | 37 (19) | Not conducted |
|  | Pearl 2003 | ‘MOPS points were an important part of my decision to take part’ survey item as an important factor in deciding to participate, total number responding | Number (%) of GPs answering | 59 | Strongly disagree 6 (10.2)  Disagree 15 (25.4)  Not sure 15 (25.4)  Agree 15 (25.4)  Strongly Agree 8 (13.6) |  |
| Doctor-patient relationship | Brodaty 2013 | ‘Improve doctor–patient relationship’ survey item as motivating factor for (possible) participation, total number responding | Number (%) of GPs answering in treatment, control, refuser groups and total | Treatment 10  Control 10  Refuser 10  Total 30 | Treatment 3 (30)  Control 3 (30)  Refuser 3 (30)  Total 9 (30) | Not conducted |
| Personal relationship with the researcher | Brodaty 2013 | ‘Personal relationship with researcher’ survey item as motivating factor for (possible) participation, total number responding | Number (%) of GPs answering in treatment, control, refuser groups and total | Treatment 10  Control 10  Refuser 10  Total 30 | Treatment 0 (0)  Control 0 (0)  Refuser 2 (0)  Total 2 (7) | Not conducted |
| Helping patients | Brodaty 2013 | ‘Help my patients further’ survey item as motivating factor for (possible) participation, total number responding | Number (%) of GPs answering in treatment, control, refuser groups and total | Treatment 10  Control 10  Refuser 10  Total 30 | Treatment 5 (50)  Control 8 (80)  Refuser 8 (80)  Total 21 (70) | Not conducted |
|  | Gunn 2008 | ‘it helped my own patients’ survey item as degree to which this influenced decision to participate, total number responding | Number (%) of GPs answering survey item by very, quite, a little, not at all agreement. | 29 | Very 17 (59)  Quite 10 (34)  A little 2 (7)  Not at all 0 (0) | Not conducted |
|  | Gunn 2008 | ‘in time it will help patients elsewhere’ survey item, total number responding | Number (%) of GPs answering survey item by very, quite, a little, not at all agreement. | 29 | Very 10 (34)  Quite 14 (48)  A little 3 (10)  Not at all 2 (7) | Not conducted |
| Reflecting on and improving practice | Brody 2013 | ‘Reflect on my practice’ survey item as motivating factor for (possible) participation, total number responding | Number (%) of GPs answering in treatment, control, refuser groups and total | Treatment 10  Control 10  Refuser 10  Total 30 | Treatment 5 (50)  Control 3 (30)  Refuser 3 (30)  Total 11 (37) | Not conducted |
|  | Gunn 2008 | ‘it allowed me to reflect on the way I practise’ survey item as degree to which this influenced decision to participate, total number responding | Number (%) of GPs answering survey item by very, quite, a little, not at all agreement. | 29 | Very 16 (55)  Quite 10 (34)  A little 3 (10)  Not at all 0 | Not conducted |
|  | Ellis 2007 | ‘Interested in improving my clinical practice’ survey item as reason for participating, total number responding | Number (%) of GPs answering | 184 | 157 (81) | Not conducted |
|  | Ellis 2007 | ‘Invigorate my practice with new ideas’ | Number (%) of GPs answering | 184 | 82 (42) | Not conducted |
| Research interest | Brodaty 2013 | ‘Interest in the research question/area’ survey item as motivating factor for (possible) participation, total number responding | Number (%) of GPs answering in treatment, control, refuser groups and total | Treatment 10  Control 10  Refuser 10  Total 30 | Treatment 3 (20)  Control 3 (30)  Refuser 2 (20)  Total 8 (27) | Not conducted |
|  | Gunn 2008 | ‘I provided the research team with knowledge and expertise from the “real world” of general practice’ survey item as degree to which this influenced decision to participate, total number responding | Number (%) of GPs answering survey item by very, quite, a little, not at all agreement. | 29 | Very 7 (24)  Quite 11 (38)  A little 6 (21)  Not at all 5 (17) | Not conducted |
|  | Ellis 2007 | ‘Like to remain involved in research initiatives’ survey item as reason for participating, total number responding | Number (%) of GPs answering | 184 | 61 (31) | Not conducted |
|  | Ellis 2007 | ‘Interested in contributing to primary prevention for coronary vascular disease’ survey item as reason for participating, total number responding | Number (%) of GPs answering | 184 | 106 (54) | Not conducted |
| Update knowledge | Brodaty 2013 | ‘Desire to gain/update knowledge and clinical skills’ survey item as motivating factor for (possible) participation, total number responding | Number (%) of GPs answering in treatment, control, refuser groups and total | Treatment 10  Control 10  Refuser 10  Total 30 | Treatment 8 (80)  Control 6 (60)  Refuser 9 (90)  Total 23 (77) | Not conducted |
|  | Gunn 2008 | ‘I updated my knowledge’ survey item as degree to which this influenced decision to participate, total number responding | Number (%) of GPs answering survey item by very, quite, a little, not at all agreement. | 29 | Very 14 (48)  Quite 13 (45)  A little 1 (3)  Not at all 1 (3) | Not conducted |
|  | Gunn 2008 | ‘I learnt new clinical skills’ survey item, total number responding | Number (%) of GPs answering survey item by very, quite, a little, not at all agreement. | 29 | Very 14 (48)  Quite 13 (45)  A little 2 (7)  Not at all 0 | Not conducted |
| **Trial Factors** | | | | | | |
| Distance to research centre | Durham 1991 | Close to clinic, yes/no | Number of patients giving consent n (%) | Not reported | Yes 1253 (57.2)  No 1462 (51.9) | Chi-squared significant at p=0.001 |
| Inclusion criteria | Fletcher 2010 | Relaxation of inclusion criteria | Recruitment rate per 1000 population | N/A | Not reported | Significant increase in recruitment rate in the last 6 months.* |
|  | Durham 1991 | Spouse invited | Number of patients giving consent n (%) | Not reported | Yes 842 (58.6%)  No 1,873 (52.4%) | Chi-squared significant at p=0.001 |
|  | Rogers 2014 | Invited as a couple or individual (number invited) | Number of patients giving consent n (%) | Couple; 471  Individual; 517 | Couple; 149 (50.0)  Individual; 149 (50.0) | OR (95% CI), p-value  Couple 1.0  Individual 0.88 (0.67, 1.15), p = 0.34 |
| Patient Recruitment method | Warren 2014 | Opportunistic (Research approaching patients in waiting room) vs Systematic (GP selecting from list of potentially eligible patients). Number of patients | Time to participant recruitment (number of days from date of recruitment of first participant to recruitment of final participant) | Opportunistic n = 73  Systematic n = 58 | Opportunistic 31.8  Systematic 86.7 | Between-group difference, mean (95%CI)  -54.9 (-103.6; -6.2) |
|  | Markun 2016 | Case-finding approach | Number (%) of patients recruited by case-finding | N/A | 71 (32.9%) | Not stated |
| Practice Recruitment method | Colwell 2012 | Viral Marketing Approach vs usual recruitment | Number of consenting GP practices  Denominator number approached | N/A | 36 (34%)  11 (24%)  7 (17%) | Nil |
|  | Ellis 2007 | 11 recruitment strategies | Recruitment rate, n (%) | 1. Medical society inserts 5350  2. Published article 3500  3. Conference distribution 106  4. Mass fax 3882  5. Minority provider direct mail 319  6. Opinion leader email 98  7. In-person provider presentation 35  8. Cold calls 318  9. In-person practice presentation 12  10. Previous relationship 27  11. AHEC survey 176 | 1. 1 (0.02)  2. 0 (0)  3. 0 (0)  4. 13 (0.33)  5. 3 (0.94)  6. 1 (1.02)  7. 0 (0)  8. 17 (5.35)  9. 5 (41.67)  10. 9 (33.33)  11. 19 (10.80) | Nil |
| Randomisation method | Warren 2014 | Cluster vs individual, number of patients | Time to patient recruitment, days | Cluster n = 102  Individual n = 29 | Cluster 58.1  Individual 58.0 | Between group difference mean (95% CI)  0.1 (-63.3; 63.5) |
|  | Brealey 2007 | Postal or telephone, number of patients recruited by either method. | Number of patients recruited per practice (median) | Telephone, n = 322  Postal, n = 231 | Telephone 2.5  Postal 1.5 | Negative binomial regression model  Coefficient and p-value  -0.147; p = 0.384  e^b (95% CI)  0.863 (0.620 to 1.202) |
| Trial Management | Fletcher 2010 | Changed approach to trial retention | Recruitment rate per 1000 population | N/A | Not reported | Significant increase in recruitment rate in the last 6 months.* |
|  | Fletcher 2010 | Tighter time-frames | Recruitment rate per 1000 population | N/A | Not reported | Significant increase in recruitment rate in the last 6 months.* |
| Financial Incentive | Jennings 2015 | £100 incentive to patients, offered/not offered. | Number (%) of patients consented and randomised | Offered 84  Not offered 97 | Offered 26 (30.9)  Not offered 24 (24.7) | Not conducted on SCOT trial. |
|  | Brodaty 2013 | ‘Receive Medicare payments for my patients’ 75+ health assessments at $171 or $200 per assessment’ survey item as motivating factor for (possible) participation, total number responding | Number (%) of GPs answering in treatment, control, refuser groups and total | Treatment 10  Control 10  Refuser 10  Total 30 | Treatment 2 (20)  Control 3 (30)  Refuser 1 (10)  Total 6 (20) | Not conducted |
| Other incentives | Ellis 2007 | ‘Wanted to receive hand held computer (personal digital assistant—PDA)’ survey item as reason for participating, total number responding | Number (%) of GPs answering | 184 | 30 (15) | Not conducted |
|  | Ellis 2007 | ‘Wanted to receive automated blood pressure device’ survey item as reason for participating, total number responding | Number (%) of GPs answering | 184 | 18 (9) | Not conducted |
| Practice Support | Williams 2014 | GP received follow-up within 2 weeks from initial training. | Rate of patients successfully recruited to the trial by each GP | 24.1% | Summary data not reported  ‘recruitment 2.2 times greater (95% CI: 1.6, 2.9; P < 0.0001)’ | Univariate analysis, coefficient (95% CI); p value  0.99 (0.52, 1.47); p <0.001  Multivariate analysis, IRR (95% CI)  2.15 (1.58, 2.94) |
|  | Williams 2014 | GP was contacted by research assistant at least once per month | Rate of patients successfully recruited to the trial by each GP | 18.9% | Not reported | Univariate analysis, coefficient (95% CI)  0.27 (-0.34, 0.89)  Not independently associated |
|  | Richardson 2002 | Practice nurse help with the trial | 1 or more patients enrolled vs no patients enrolled. | Yes 46 (48)  No 49 (52) | Recruited for the trial  Yes 26 (56%)  No 16 (33%) | Chi-squared = 5.48, p = 0.02 |
|  | Fletcher 2010 | Workload reduction for practices | Recruitment rate per 1000 population | N/A | Not reported | Significant increase in recruitment rate in the last 6 months.* |
| Involvement of academia | De Wit 2001 | ‘motivation by the participation of the academic research group’ survey item as a motivating factor for participation | Number of patient recruited | Data tables unavailable | Data tables unavailable | Adjusted OR 2.9 (95% CI = 1.2-6.9) |
| Professional endorsement | Brodaty 2013 | ‘Endorsement by RACGP and/or division’ survey item as motivating factor for (possible) participation, total number responding | Number (%) of GPs answering in treatment, control, refuser groups and total | Treatment 10  Control 10  Refuser 10  Total 30 | Treatment 2 (20)  Control 0 (0)  Refuser 2 (20)  Total 4 (13) | Not conducted |

*****Fletcher 2010 conducted a time-series analysis which reported the results for multiple factors that were changed at the same time. It is therefore important to conclude that nothing can be inferred about the effect of any individual factor.
