# Supplementary material for: Factors associated with recruitment to randomised controlled trials in general practice: a systematic mixed studies review
Source: Trials. 2023 Feb 6;24:90. doi: 10.1186/s13063-022-06865-x (PMC9903494; doi:10.1186/s13063-022-06865-x)
Supplement: Supplementary file 7 — Additional file 7. Qualitative results tabulation. [file 13063_2022_6865_MOESM7_ESM.docx]

| **Recruitment of patients** | | |
| --- | --- | --- |
| Patient factors | | |
| Higher order theme | Subtheme | Illustrative quotes |
| Medical factors | Desire not to have medication changed | **‘***My medicines are all right and I don’t want them mucking about.*  *‘I’ve been on this for years and I’ve been alright’ [1]*. |
|  | Current health status | Health problems could directly affect participation through limited mobility in a physical activity trial, ‘*I had the stroke in ’94. So that limited my walking*’ [2].  The additive burden of multimorbidity could also affect participation,  ‘*I don’t need anything else going on to do with health … I certainly would have thought … that they would have thought, “oh, she wouldn’t want to do this because she’s got lots of other problems*”. [2]  ‘*psychological barriers*’ were reported by some to affect participation’, ‘*I suffer a lot of pain all the time, 24 hours a day, and it gets me depressed’* [3]*.* |
|  | Fear of health risks | Perceived negative effect of the intervention due to implications of an ‘emergency card’, ‘*initially I said yes (...) I would then have to have this emergency card. So that if something happened, so they knew what I had taken. Then (...). I panicked, no, I don’t want this, I don’t want it (...) Because I only know about these [emergency cards] from patients taking Marcumar* (anticoagulant) *or diabetics (…), and there it’s a matter of life and death.* [4]’  Negative effect due to have to increase activity,  ‘*I think that was one of my main worries, that I just felt my knee would get worse*. [3]’  Perceived risk of the control group’s condition worsening due to not being treated, ‘*… just that people who are sick anyway, do not want to risk worsening of their condition … you go to the doctor because you want to make it better.* [4]’ |
| Practical factors | Time constraints | Time as a barrier to participation in research generally,  ‘*In all fairness there was no decision not to attend, I wanted to do it…and then things get put to one side…I was busy with work’* [5]*.*  Time as a barrier to specific interventions e.g. activity, ‘*I work nine to five in an office and then come back home and I’ve got two children and my husband…. So then by the time I finish with them it will be like half past eight or eight o’clock and then I feel too tired to…’* [5].  Participants thought that some did not take part due to the time required, especially for employed people, ‘with more complex, time-consuming studies, they simply do not have the time to attend’ [4]  Some had many other commitments relating to work, family and recreation, ‘*I’m just really rather busy, I play a lot of sport and look after various people and I just don’t feel I would have the time to fit it all in’* [3].  A long duration as a barrier, ‘*I think 3 months is a bit of a long time.’* [3].  ‘*It does sound a bit on the lengthy side doesn’t it really… some people could be put off by that’*[2]. |
|  | Travel difficulties | “*If I had time, I’d love to be part of your research and go to the surgery and all the rest of it, but I think, actually … the awkwardness of the journey…”[2]*. |
|  | Work and other commitments | One non-participant described difficulty in getting time off work, ‘*It’s bad enough trying to get … a day off for a normal appointment*’ [2].  Another described a variety of competing demands, ‘*I’ve got grandchildren. I’ve got a husband. I like to do my gardening. I’ve got a four bedroom house to keep clean. I feel my load is more than enough to keep me going*’ [2].  Caring commitments were also found to deter participation, ‘*I’m a carer for my father. I think most of it is just being there’* [2]*.* |
|  | Travel away | *‘I’m going away so much, I couldn’t really tie myself down to anything like that’* [2] . |
| Beliefs | Candidacy | Active enough, ‘*I already do run about four times a week and cycle to and from work…and do Pilates, so I probably wouldn’t increase it. I felt like probably I wasn’t the ideal candidate’*[5].  ‘*I don’t really think it was a necessary thing for me to do because I think I do a lot of exercise anyway.’* [3]  *Too young,* ‘*I just remember thinking, actually, I don’t think I’m in that age group yet. It kind of seemed to be geared to people who really were in their 70s and over*’ [2].  *Perceived simplicity of medication regimen, ‘*‘*would be wasting the pharmacist’s time*’, ‘*I only take Aspirin*.’[1]  On the advice of others, ‘*I did mention it to my daughter actually and she said “that sounds crazy!*” *She said it’s not for me, so I didn’t go any further’*[2]. |
|  | *Perception of research as an experiment*  *The value of research* | ‘*fear … that one would be subjected to some kind of medication ... that you would somehow be used as a guinea pig’* [4].  *Not valuing research, ‘…because many think, yes give me the medication and I’ll go home and then just leave me in peace’ [4].* |
|  | *Mistrust of the study* | ‘*Is it to change my medicines to a cheaper one, is that what it's all about?*’ [1]. |
|  | *Attitude toward healthcare* | *Did not like contact with the healthcare system, ‘They (the health care system) are not interested when you get old’* [1]. |
|  | *Media portrayal of research* | ‘*this is also quite a large theme in the media, because it is always portrayed as very negative, or immediately some kind of drama is made out of it*’ [4]. |
|  | *Perceived self-efficacy* | ‘*Exercise is in my own hands if I choose to do something about it as opposed to being part of an organised team to do it’ [3]* |
|  | Too old for exercise | ‘*I find an awful lot of people our age, they think about exercise but very few do anything about it. As they get older, they slow down and can’t be bothered’ [3].* |
| Behaviours | Altruism | Helping research in general, ‘…*without people who participate research just can’t be done…if research is not done, then you can’t find out anything. And if no one participates in such studies, then that’s a problem*. [4]’  ‘*I would like to think of a way to help them in any way, research or whatever’* [5].  Helping people with specific conditions, ‘‘*(...) you have to do your part so that such studies can be run. If there really is the possibility that you might protect patients from always having to take antibiotics, (…) then you have to do your part*.’ |
|  | *Spontaneity* | ‘I spontaneously declared my readiness. As such, I have not given it much thought before. I was just being open and curious.’[4] |
|  | *Inflexibility to changes in existing routine* | ‘*I've got into a routine I like to stay in. If I go and do anything different, it sounds silly, but I get a bit uptight*" [3]. |
|  | Research experience | “…*because I’ve already participated in such a study, I said, okay, this is for me ... if something like that is running, I’ll take part.*”[4] |
| Attitudes | Gratitute for a medical assessment | ‘*And the nurse asked me to participate... and because I was glad I got to see someone at all, I naturally agreed*.’[4] |
| Practice Factors | | |
| GP surgery-related barriers | Access to the practice | ‘*But just no one would pick the phone up at the surgery for me to actually book on*’ [5]. |
|  | Dislike of attending the practice | ‘*I have to go there when I’m not well. I certainly am not going to go to the surgery when I’m well*’ |
| Practitioner Factors | | |
| Doctor-Patient relationship | Not wishing to undermining relationship | ‘*I trust my GP (to give me the right medicines) I don’t want him to think I’ve come complaining to you (the pharmacist)*’ [1]. |
|  | Patient-doctor trust | Trust in the GP’s recommendation of the trial ‘Yes, basically I have trust in my doctor. I feel in good hands…therefore I had no concerns that they would try anything or that something bad would happen to me’ [4].  Communication with the GP reassuring some more than the trial information sheet, ‘*And he assured me that nothing bad happens, that the drugs that are used are not any old experimental laboratory drugs(…). I do not know if I would have readily decided [to participate] without a discussion with the [GP].* [4]*’*  GP conviction in the trial influencing the decision, ‘*As a result, she [GP] had actually convinced me, because she was convinced of it [ICUTI trial] herself*. [4]’ |
|  | Forgetfulness | Due to incident recruitment, ‘‘*This is a proactive study, therefore I have to remember it at the moment itself*’ [6].  Due to low number of eligible patients, ‘*Low amount of eligible patients. Because of this, I don’t think about including a patient in the trial at the right moment; I forget it*’ [6].  Addition of a note in the patient record was suggested, ‘*The GP assistant is the first contact moment with the patient; it would already help if they ... put a note in the patient’s file about the possibility of participating in the trial’* [6].  As were reminder emails, *‘It probably would have helped to have a few reminder emails. I tried to make myself remember by putting up a sign at eye level beside my computer, but that didn’t help’* [7]. |
|  | Confusion about the recruitment strategy | Leading to failure of implementation, ‘*All the documentation and stamps arrived. My understanding was that your researcher or office would contact our office manager to explain the study. This did not occur. I was left without direction’* [7]. |
|  | Time pressures | Relating to incident recruitment, ‘*I think in any trial . . . there’s never a good time to pick out patients in the middle of a busy surgery . . . and sometimes that can put a bit of pressure on you’* [8]. ‘*I intended to try to recruit patients but I think there is too much going on during the consultation to assess LBP that to add to it by discussing a ‘study’ would have been information overload. So unfortunately, recruiting for your study was the first thing to go - just not enough time for everything in general practice’* [7].  Due to competing demands, ‘*Attention in our general practice is mostly claimed by countless organisational tasks and duties like accreditation, new legislations (recently two new laws) and so on. We can’t handle more than sporadic and ad hoc duties’* [6]. |
|  | Number of eligible patients | Fewer patients with URTI, ‘*I get the impression that there are less kids with colds basically coming to surgery than there used to be. Whether that’s our success in educating them . . . or whether that’s just changes in society, I don’t know’* [8].  Restrictive eligibility criteria, ‘*The inclusion criteria were very specific and narrow; especially concerning the pain score’* [6]. |
|  | *Randomisation concerns* | Conflict between shared decision-making and randomisation to treatment, ‘*Usually the treatment of choice is the result of a — sort of — negotiation between patient and GP…Often patients already used paracetamol, or react like: “Paracetamol?”, and they feel unheard*’ [6]. ‘*Some GPs specifically mentioned that since they discuss the need for sick leave thoroughly with their patients, and subsequently arrive at a joint decision, sick leave could not be based on randomization’* [9].  Negative impact on doctor-patient relationship through something being left to chance, ‘*To participate in research where sick leave was a result of randomization would demonstrate lack of trust in the GP’s judgment, as well as the patient’s confidence in their ability to work.’* ‘*A lot of patients will probably be angry and feel they are not taken seriously if I suggest randomization to decide their need for sick leave. I wish to preserve a good relationship with my patients, both on a professional and human level*’ [9].  Since sick leave was a right, it should not be randomised, ‘*To a large extent the patients perceive sick leave as a right they can demand*’ [9].  GPs made their own decisions about what was in the best interests of the patient, ‘*Faced with a patient, in your own mind you’ve made a therapeutic decision one way or the other: either they need counselling or they don’t’* [10]. ‘*I felt uneasy about being able to manage the patient without recourse to counselling’* [10]. ‘I sick list patients who need sick leave and I can’t just start randomizing’ [9].  Patients also having their own judgements about what is in their best interests, ‘*…the major difficulty was asking patients most of whom came with pre-conceived ideas about what they want*. [10]’  Concerns due to |
|  | Concerns about delayed treatment | ‘*The trial concerns a group of patients that demands and needs direct help. The research setting then causes an unnecessary and unwelcome delay’* [6]. |
| Trial Factors | | |
|  | Patient recruitment method | Reluctance to recruit less well patient to the trial ( incident recruitment) ‘*if they were quite poorly I wouldn’t be putting them into CHICO, because it would take me um… longer to do that consultation and examination and think about the plan of care.[11]*’  Practices with systems in place to channel patients to higher recruiters increased recruitment, ‘*in a busy duty surgery, whilst triaging, would just forget and would book them in as the normal route through the duty surgery or with a nurse practitioner. So there were definitely probably children being seen that were missed.*’ |
|  | Trial invitation material | The length of the trial material as a reason for declining, “*… there was a lot to read. Bullet points are good. Just make it simple*” [2].  Difficulties in the ‘*readability and comprehension’* of the invitation letter, ‘*I had difficulty reading the letter and I had no one to read it for me’* [1]. |
|  | *Confusion or lack of understanding of the trial* | Many respondents were unclear of the details of the trial, ‘*The majority of interviewees only vaguely recollected receiving letters and did not appear to pay attention to their content’ [5].*  Two respondents emphasised that some will not read through the participant information in detail, ‘*Just a health check. I don’t really know what it would have involved’*; ‘*I can’t actually remember it, to be honest, but I probably didn’t think about it that much’ [5]*.  Several non-participants had not understood several aspects of the trial.  ‘Some infirm or housebound patients failed to understand that they could be visited at home. “*I can’t get down to the surgery. I do not go out”.* [1]*’*  ‘Other patients thought the study was a clinical trial involving experimental medicines despite the letter explicitly stating it was not this type of study. [1]’  **‘**Confusion arose over the meaning of words. “*I don’t take any medicines”*. Their definition of a medicine was an oral liquid, tablet or capsule. Creams, inhalers etc were not considered to be “medicines”. [1]’  ‘Cognitive impairment resulting in confusion was apparent for some patients who had failed to reply and were phoned. There was also some misunderstanding due to deafness... [1]’ |
|  | *Randomisation concerns* | Not knowing which treatment would be received, ‘*And that ... was what really stopped me. I am willing to participate in studies ... but if I do not know what I am taking then I am not so supportive.* [4]*’*  Preference for specific management, ‘*If I had had a massive urinary tract infection and had I said “I need something now”, then I would have participated.’* [4]  *‘I think if you’re doing research then you should be able to choose …within reason …what club you’re willing to join really’* [2].  Reluctance to be in the control group, *‘Well … I couldn’t see the point of being in a group that did nothing’* [2]. |
|  | Intervention unappealing | Reluctance to walk alone, in the evening, or in poor weather, ‘*It’s a bit off-putting I think really, as it’s very wet and gets dark early -you don’t want to be walking in it.*’ [3]  ‘*As the weather gets better, then I might go for a walk in the evening … it was really due to the seasons as well’* [2].  Lack of interest in exercise, ‘*I don’t want to spend every minute I’ve got free thinking I’ve got to do exercise*’ [3].  ‘*Walking’s quite boring. Unless you’re walking somewhere on an outing somewhere, you know, in the country or something, seaside. You should have more trips*’[2].  Concerns regarding use of the accelerometer due to perceived discomfort, ‘*It would put me off a bit because I’m waiting to have a heart monitor - I can’t cope with two*’, and problems with recording, ‘*The monitor only indicates steps and I have problems with my feet so I ride a bike and that doesn’t record so well on the monitor*’ [3].  Use of a pedometer, ‘W*ell, I mean I have actually used a pedometer but I wouldn’t sort of particularly want to do it for a week’* [2].  Mixed preference for one-to-one consultations over group consultations, ‘*I’m not very good in groups I would say. I think I would prefer one-to-one*’ [3]. ‘*I think you get more encouragement if you are in a group*’ [2]. |
| Practitioner perception of factors associated with recruited | | |
|  | Lack of patient incentives | ‘*Suggest a free physiotherapy appointment/or better still a $30 petrol voucher for their time if complete survey. You would be inundated*.’[7] |
| Recruitment of Practices and Practitioners | | |
| Practice factors | | |
|  | Decision made on behalf of practices and practitioners | Decision made by managers negatively influencing recruitment, ‘*These studies are so important to the discipline, but what makes sense for the time and energy of the clinicians and entire clinic unit is still a bit unknown…[Our practice] is doing great work in terms of PCMH [Patient Centered Medical Home] and is leading the pack in so many ways—we want to make sure we guide them about what to expect if they participate in the future’ [12].*  ‘*Once we feel that the practices are comfortable, we will turn them over to you to get the process rolling*’ *[12].*  ‘*We are not a good source of practices for this study given the composition and interests of our practice population*’ *[12].* |
|  | Ease for the practice | Motivated by the ease with which their practice could participate, ‘*Um ... we have a very mixed population and we have a phlebotomist on site, it’s very easy for us to offer this to a patient and then they can quite easily get an appointment, it’s not too much hassle for them. And we do see a lot of pregnant women, a lot…’* [13]. |
|  | *Research interest and perception of research value* | a ‘*special interest’* in the research topic could increase motivation to participate, ‘*I understand the value of clinical research, but honestly the clinical staff at our program are so busy that only two things would get us involved, 1. special interest in the topic (which no one here has)*’ [12]. The second was financial which is included in the relevant trial factor section.  Few GPs wishing to be involved or learn about research, ‘*only five GPs mentioned their desire to contribute to or learn about research’* [14].  Some GPs wished to participate due to the ‘perceived intrinsic value of research’, *‘I’m quite keen to know what’s going on. I think it’s important, so I’ve got no problems with that. [8]’*  *‘I thought it was a scientifically very interesting idea. Super idea. I’m very keen on taking advantage of people’s ideas when they suddenly notice something and to be able to build it up and help it come to fruition. [8]’* |
|  | Time Constraints | High workload of health care organisations impacting on participation, ‘*I have four current projects I am working on … and I just do not feel I can commit to another right now’* [12]. ‘*We are just so busy so the board decided it is too much and docs will be running screaming from the building if we ask them for one more thing to do*’.  ‘*The workload has quadrupled over the last 20 years, mainly due to the endless contract box ticking, non-contract box ticking, more administration and more patient demand.* [15]’ *‘Well I would simply say I have no time, I can’t take part in the research. We’ve got no time, lack of time. It’s basically a time factor, no time’* [16].  Requirement for the trial to fit well with existing workflow given time pressures, ‘*It depends how difficult the study is and how much it messes up the way we work*’ [15].  Low perceived time-pressures of the SAVIT study were found to be a positive aspect, ‘*well, I mean, you’ve only got so much spare time and you know, if it’s taking half an hour a patient, . . . but if you’ve got two in a morning I mean, it makes life just miserable’* [8]. |
| Trial Factors | | |
| Clinical Relevance | Need for research to be clinically important to practice population | ‘*Most GPs also identified the need for the research to be locally relevant and clinically important for the local population*’ [17]. ‘*Because there’s no active treatment for viral infection and I mean children get very distressed and miserable don’t they and they’re crying all the time and it’s just something to try and see if it will improve their symptoms over and above Calpol basically’* [8]. ‘*Mainly because we thought it was a good idea because our population we serve is a very ethnic mix and we see a lot of Afro–Caribbean, African, patients and Middle Eastern patients as well, so we thought it would be good for the women to find out early on what their status was’* [13].  GPs preferred ‘*research relevant to their role at the ‘coal face’ of dementia services’* [15]. |
| Participant recruitment method | Opportunistic recruitment causing additional time pressures | ‘*Surgeries are already long and gruelling so only two GPs in the practice were willing to add an extra 5 minutes into their consultations for another study using hot recruitment.* [17]’  *‘Time is a fundamental problem.* [17]*’* |
| Perceived ease of recruiting patients | Computer-based pop-up alerts | As a negative influencing factor, ‘*the partners and GPs are not happy with additional pop ups, they’re clutter and an aggravation factor*’ [17].  As a positive influencing factor, ‘*I liked LEPIS to reduce the workload element of identifying patients through searches and getting a timely reminder removes the stress of having to remember the study all the time’* [17]*.* |
|  | Number of eligible patients | low incidence of COPD and the impact of pre-existing rescue packs on eligible numbers, ‘*We have a very low incidence of smoking and COPD so it’s not a priority issue and not worth the effort. It’s 20 years out of date as well, now we have rescue packs and micro-manage patients on the basis of need. It’s not very practical.* [17]’ |
| Incentives | Financial incentives | healthcare organisations states that clinicians may participate if ‘*enough money*’ was provided to generate interest, ‘*The study sound intriguing…for us to get providers involved there need to be some carrot*.’ [12]. ‘*The payment’s the ﬁrst thing you look at basically. Is it worth me looking any further and if it is then you look further ... or you might not, I can’t do the time, I’m not interested’* [16].  Clinicians appreciated remuneration but that it was not a major factor in participation, ‘*they appreciated remuneration but did not rank this highly’* [15]*.*  *Mixed-views on making a profit, ‘It’s important to make a profit for income as well as cover costs.*’  *‘Remuneration from [other organisations] has not been adequate, primarily because small numbers of patients were identified for the study. We don’t expect to make a profit but we’re not happy to run at a loss.*’  ‘*To be honest you will only get GPs to do it if you give them some ﬁnancially make it worth their while because they will say that their time is precious, that their time is their spare time that they want to do something else, you know even if it’s just going out with the family, whatever. They will say they should be getting paid for it*’ [16]. |
|  |  |  |

1. Petty DR, Zermansky AG, Raynor DK, Vail A, Lowe CJ, Freemantle N, Buttress AD: **"No thank you": why elderly patients declined to participate in a research study**. *Pharm World Sci* 2001, **23**(1):22-27.

2. Normansell R, Holmes R, Victor C, Cook DG, Kerry S, Iliffe S, Ussher M, Fox-Rushby J, Whincup P, Harris T: **Exploring non-participation in primary care physical activity interventions: PACE-UP trial interview findings**. *Trials [Electronic Resource]* 2016, **17**:178.

3. Rogers A, Harris T, Victor C, Woodcock A, Limb E, Kerry S, Iliffe S, Whincup P, Ekelund U, Beighton C *et al*: **Which older people decline participation in a primary care trial of physical activity and why: insights from a mixed methods approach**. *BMC geriatrics* 2014, **14**:9.

4. Bleidorn J, Bucak S, Gagyor I, Hummers-Pradier E, Dierks ML: **Why do - or don't - patients with urinary tract infection participate in a clinical trial? A qualitative study in German family medicine**. *German Medical Science* 2015, **13**:Doc17.

5. Attwood S, Morton KL, Mitchell J, Emmenis MV, Sutton S, Team VBIP: **Reasons for non-participation in a primary care-based physical activity trial: a qualitative study**. *BMJ Open* 2016, **6**(5):e011577.

6. van der Gaag WH, van den Berg R, Koes BW, Bohnen AM, Hazen LMG, Peul WC, Voogt L, Verhagen AP, Bierma-Zeinstra SMA, Luijsterburg PAJ: **Discontinuation of a randomised controlled trial in general practice due to unsuccessful patient recruitment**. *BJGP Open* 2017, **1**(3).

7. Page MJ, French SD, McKenzie JE, Connor DAO, Green SE: **Recruitment difficulties in a primary care cluster randomised trial: investigating factors contributing to general practitioners' recruitment of patients**. *BMC medical research methodology* 2011, **11**:35.

8. Prout H, Butler C, Kinnersley P, Robling M, Hood K, Tudor-Jones R: **A qualitative evaluation of implementing a randomized controlled trial in general practice**. *Family practice* 2003, **20**(6):675-681.

9. Maeland S, Magnussen LH, Eriksen HR, Malterud K: **Why are general practitioners reluctant to enrol patients into a RCT on sick leave? A qualitative study**. *Scand J Public Health* 2011, **39**(8):888-893.

10. Fairhurst K, Dowrick C: **Problems with recruitment in a randomized controlled trial of counselling in general practice: causes and implications**. *J Health Serv Res Policy* 1996, **1**(2):77-80.

11. Blair PS, Turnbull S, Ingram J, Redmond N, Lucas PJ, Cabral C, Hollinghurst S, Dixon P, Peters T, Horwood J *et al*: **Feasibility cluster randomised controlled trial of a within-consultation intervention to reduce antibiotic prescribing for children presenting to primary care with acute respiratory tract infection and cough**. *BMJ Open* 2017, **7**(5):e014506.

12. Loskutova NY, Smail C, Ajayi K, Pace WD, Fox CH: **Recruiting primary care practices for practice-based research: a case study of a group-randomized study (TRANSLATE CKD) recruitment process**. *Family practice* 2018, **35**(1):111-116.

13. Dormandy E, Kavalier F, Logan J, Harris H, Ishmael N, Marteau TM, Anionwu EN, Atkin K, Brown K, Bryan S *et al*: **Maximising recruitment and retention of general practices in clinical trials: A case study**. *British Journal of General Practice* 2008, **58**(556):759-766.

14. Gunn J, McCallum Z, Sanci L: **What do GPs get out of participating in research? - experience of the LEAP trial**. *Australian family physician* 2008, **37**(5):372-375.

15. Brodaty H, Gibson LH, Waine ML, Shell AM, Lilian R, Pond CD: **Research in general practice: a survey of incentives and disincentives for research participation**. *Ment Health Fam Med* 2013, **10**(3):163-173.

16. Salmon P, Peters S, Rogers A, Gask L, Clifford R, Iredale W, Dowrick C, Morriss R: **Peering through the barriers in GPs' explanations for declining to participate in research: the role of professional autonomy and the economy of time**. *Family practice* 2007, **24**(3):269-275.

17. Staa TPv, Dyson L, McCann G, Padmanabhan S, Belatri R, Goldacre B, Cassell J, Pirmohamed M, Torgerson D, Ronaldson S *et al*: **The opportunities and challenges of pragmatic point-of-care randomised trials using routinely collected electronic records: evaluations of two exemplar trials**. *Health technology assessment (Winchester, England)* 2014, **18**(43):1-146.
