# Supplementary material for: Factors associated with recruitment to randomised controlled trials in general practice: a systematic mixed studies review
Source: Trials. 2023 Feb 6;24:90. doi: 10.1186/s13063-022-06865-x (PMC9903494; doi:10.1186/s13063-022-06865-x)
Supplement: Supplementary file 8 — Additional file 8. Glossary. [file 13063_2022_6865_MOESM8_ESM.docx]

**Glossary**

AXIS Appraisal tool for Cross-Sectional Studies

B-coefficient logarithm of e^B and interpreted as a multiplying factor when exponentiated

Candidacy A theoretical framework based on ‘*lay epidemiology*’

Celecoxib A cyclo-oxygenase-2-inhibitor NSAID

CASP Critical Appraisal Skills Programme

CI Confidence Interval

Cluster RCT RCT where the randomisation unit is the general practice

CME Continuing Medical Education

COPD Chronic Obstructive Pulmonary Disease

disodium anti-inflammatory/anti-allergy agents

cromoglycate

DistillerSR Systematic review software

EOI Expression of interest

e^B the unit change in the dependent variable for every unit increase in the independent variable

GP General Practitioner

H. pylori Helicobacter pylori – digestive tract bacteria that can cause dyspepsia.

HTA Health Technology Assessment

ICUTI Immediate vs. Conditional Use of Antibiotics in Uncomplicated Urinary Tract Infection

IPA Independent Practitioner Association

IRR Incident Rate Ratio

LBP Low Back Pain

LEPIS Local Eligible Patient Identification Service

NIHR National Institute for Health Research

NSAID Non-steroidal anti-inflammatory drug

Marcumar brand name of Phenprocoumon, a vitamin K antagonist anticoagulant

MEDLINE National Library of Medicine’s (NLM) bibliographic database

NRSI Non-randomised studies of interventions

NVivo qualitative data analysis software

OpenGrey System for Information on Grey Literature in Europe

OR Odds Ratio

Ovid Medical research platform

PDA Personal Digital Assistant

PRioRiTy Prioritising Recruitment in Randomised Trials

PRISMA Preferred Reporting Items for Systematic Reviews and Meta-Analysis

PRISMA-P Preferred Reporting Items for Systematic Reviews and Meta-Analysis Protocols

PRISMA-S Preferred Reporting Items for Systematic Reviews and Meta-Analysis literature search extension

PROSPERO The International Prospective Register of Systematic Reviews

RA Research Assistant

RACGP Royal Australian College of General Practitioners

RoB 2 Cochrane risk-of-bias tool for randomized trials

ROBINS-I Cochrane Risk Of Bias In Non-randomised Studies of Interventions

RCT Randomised controlled trials

SAVIT Suspected Acute Viral Infection Treatment

SES Socioeconomic Status

URTI Upper Respiratory Tract Infection
